# An eight-camera fall detection system using human fall pattern recognition via machine learning by a low-cost android box

**DOI:** 10.1038/s41598-021-81115-9

**Published:** 2021-01-28

**Authors:** Francy Shu, Jeff Shu

**Affiliations:** 1 Division of Neuromuscular Medicine, Department of Neurology, Los Angeles Medical Center, University of California, 300 Medical Plaza B200, Los Angeles, CA 90095 USA; 2 SpeedyAI, Inc, 19940 Ridge Estate Ct, Walnut, CA 91789 USA

**Keywords:** Health care, Neurology, Engineering, Mathematics and computing, Optics and photonics

## Abstract

Falls are a leading cause of unintentional injuries and can result in devastating disabilities and fatalities when left undetected and not treated in time. Current detection methods have one or more of the following problems: frequent battery replacements, wearer discomfort, high costs, complicated setup, furniture occlusion, and intensive computation. In fact, all non-wearable methods fail to detect falls beyond ten meters. Here, we design a house-wide fall detection system capable of detecting stumbling, slipping, fainting, and various other types of falls at 60 m and beyond, including through transparent glasses, screens, and rain. By analyzing the fall pattern using machine learning and crafted rules via a local, low-cost single-board computer, true falls can be differentiated from daily activities and monitored through conventionally available surveillance systems. Either a multi-camera setup in one room or single cameras installed at high altitudes can avoid occlusion. This system’s flexibility enables a wide-coverage set-up, ensuring safety in senior homes, rehab centers, and nursing facilities. It can also be configured into high-precision and high-recall application to capture every single fall in high-risk zones.

## Introduction

A fall in this study is defined as an event in which a person suddenly and inadvertently collapses from an upright position and the person’s legs can no longer support oneself.

Worldwide, falls are a leading cause of unintentional injuries in adults older than 65 years old, with 37.3 million falls requiring medical attention and 646,000 resulting in deaths annually^1^. Seniors living alone are at high risks^1^. Many common neurological problems result in falls^2^: Peripheral neuropathy^3^ manifesting with numbness and imbalance, spinal stenosis^4^ resulting in pain and incoordination, acute strokes^5^ leading to sudden weakness, and Parkinson disease^6,7^ characterized by postural instability, etc. In addition, cardiovascular^8^, musculoskeletal^9,10^, and medication-induced^11^ problems often coexist^12^. Orthostatic hypotension^13,14^, knee arthritis^15^, and iatrogenic dizziness^12,16^ are only a few examples. Even for healthy seniors^17,18^, activities such as climbing ladders, taking showers, going down stairs, and walking in snow could be dangerous.

In fact, falls are not exclusively problems of the elderly, but may also be concerns for the young^19^. Postural Orthostatic Tachycardia Syndrome^20^, seizures^21^, anemia^22^, pregnancy^23^, and sports^24^ all can lead to unexpected falls. Without timely detection and treatment, complications such as bone fractures^25,26^, intracranial hemorrhage^25^, or nerve avulsion^27^ can result. Permanent disabilities and death are not unusual^1^. In 2015, the medical cost for falls exceeded $50 billion^28^. As the world population ages, the number of serious falls and subsequent financial burdens rise accordingly. It is imperative to detect falls timely to initiate appropriate medical responses to reduce the significant physical, social, and financial damages.

Currently, fall detection methods are broadly classified as wearable devices, environmental sensors, and image detectors, but significant limitations exist. Wearable sensors utilize tri-axial accelerometers to measure body inclination and are mounted to the wrist or another body part, or attached to the shoe insoles or garment fabrics^29–31^. Gyroscopes estimate the rotational acceleration^29^. Unfortunately, these sensors need frequent manual calibration due to fluctuations in temperature and humidity, and could send false signals. Also, people could forget or feel uncomfortable wearing these devices, or fail to replace batteries. Furthermore, healthy seniors can also fall accidentally but they usually do not have these devices, which require a costly 24-h monitoring team and monthly subscription fee.

The second detection category utilizes various environmental elements. For example, acoustic sensors measure the sound of falls^32^; pressure sensors measure the weight changes on the floor^33^. Infrared sensors map out a person’s heat signature and ultrasound detectors process the return signal^34,35^. Near-field imaging with matrices of electrodes under floors track fall patterns^36^. A common challenge is differentiating humans from animals or objects. Usually, the detection accuracy by acoustic arrays decreases if the person is five or more meters (m) away. Besides, some technologies are too expensive and impractical to be installed in every room. Even for the less costly wireless physical layer using channel state information, detection fails with multiple people in the room or if the furniture is pushed away during the fall, interfering with the mathematics designed only for single-entity monitoring^37^.

The third category is image-based and is sub-classified into multiple cameras, single cameras, or images with three-dimensional (3D) depth data.

The multiple-camera network reconstructs a 3D image, analyzes the volume distribution of the individual along the vertical axis, and triggers an alarm when most of the volume is near the floor for a predefined period^38^. This system requires a complicated setup with time-consuming calibration and fails to detect falls when there is more than one person in the room or when one is partially occluded by furniture.

The single-camera method uses fuzzy logic or machine learning to detect postural changes^39–42^. However, this approach makes fall detection along the optical axis difficult. The same challenge lies in falls partially occluded by objects such as furniture. Alternatively, deep learning is used to train video-based fall detection models^43^. One barrier for this approach is that it requires many hundreds of thousands to millions of high-quality human fall videos as training data, but such procurement is infeasible. Another barrier is its intensive computation demand. Therefore, many researchers focus on indirect fall detection based on inactive time, specifically the time one spends lying in the horizontal position. Unfortunately, this prevents the differentiation of a true fall from resting on the floor, doing yoga, and performing other lower-body exercises. Moreover, the complex mathematics and algorithms in machine learning usually require dedicated high-end hardware in a cloud computing setting, which may sacrifice user privacy. Also, the infrastructure overhead for a city-wide deployment of such a cloud-based Artificial Intelligence (AI) system would be beyond the capability of even large enterprises^44^, rendering it utterly impractical.

One published work that has one of the more cost-effective systems uses a Raspberry Pi 2 with a single camera, but the resolution was 320 by 240 pixels at 7–8 frames per second (FPS), detecting falls at up to 10 m^45^. Currently, even with the updated Pi 4 improving the resolution and frame rate, it remains a challenge for this published model to monitor a whole house. Lastly, for images with 3D depth data, either a multiple- or single-camera system is used with an infrared or ultrasound depth meter to identify the direction of the faller^46,47^. However, it can track up to only a distance of 3.5 m and fails to detect falls when more than one person is in the room. This method also misses falls in rain and shower or behind a transparent glass or plastic door.

For image and environment-based sensors, the typical cost of a system with two monitoring rooms averages $1500 excluding monthly subscription fees, making the service unaffordable for most people.

By analyzing the physics of falls, we found that a few parameters such as velocity, acceleration, and motions of the head and legs are well correlated with falls. By extracting these parameters to augment image semantics-based features, we can use any machine learning method to recognize falls. This theory allows us to use a parsimonious AI model to effect extremely efficient processing of videos at a high frame rate to combat the challenges currently faced in fall detection accuracy, computational efficiency, and financial cost. We have developed a novel measurement method with high detectability, utilizing conventionally available and inexpensive surveillance camera systems including multi-camera with multi-angle capability, without any need for complex calibration. The system can monitor eight cameras in various locations and different rooms. If a more powerful micromcomputer like Raspberry Pi 4 is used, up to twelve cameras can be monitored, reaching a detection distance of 10 m and camera resolution as low as 640 pixels by 360 pixels. For falls at much longer distances, monitoring is also possible with affordable high-resolution cameras. The proposed system can detect falls with cameras mounted from 0° (low-altitude installation) up to 45° downward angle (high-altitude installation), compatible with the security monitoring camera systems.

### Fall analysis

We first characterized the biomechanics of the most common types of falls. A fall occurs when the center of the gravity (CG) of a person’s trunk becomes misaligned with the base of support provided by the feet against the floor^48^. The CG is an imaginary point at the level of the sternum anterior to the spine, at which all the weight of the torso is evenly distributed.

**Figure 1:**
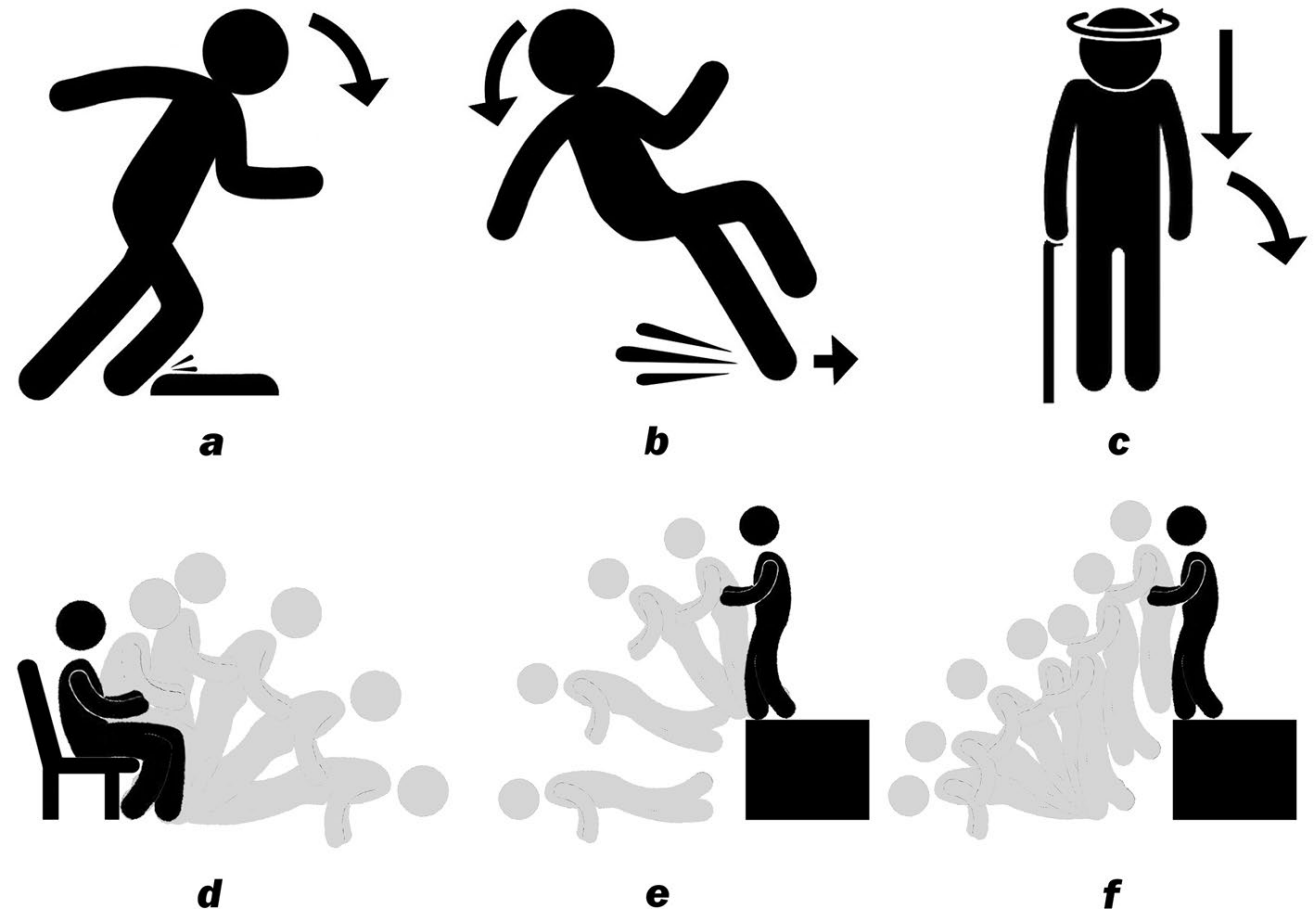
Selected fall types. (**a**) Stumbling. (**b**) Slipping. (**c**) Fainting. (**d**) Getting up from a sitting position (i.e., a chair) and falling as in orthostasis. (**e**) Falling from a high structure (i.e., stairs, ladders, etc.). (**f**) Jumping down from a high structure and falling.

Stumbling (Fig. 1a) results from accidentally stepping onto an unperceived object while inertia keeps the CG moving, resulting in an imbalance in the torso. The trunk can flex anteriorly and so the edge of support approximates a vertical line passing through the tarsometatarsal joints of the foot. When the CG is shifted beyond this edge, due to resistance encountered in the moving feet, the person stumbles and falls. Stumbling commonly occurs in poorly lit rooms with misplaced items on the floor. Individuals with neurological or musculoskeletal disorders are more at risk for stumbling.

Slipping (Fig. 1b) occurs when the frictional force opposing the direction of foot movement is less than the horizontal shear force of the foot immediately after the heel contacts the floor^49^. The legs slide out of place and the person can no longer stay upright. Seniors have a reduced density of sensorimotor nerve fibers in the feet and often slip in bathrooms and kitchens or when walking downstairs^50^. Improper footwear and environmental obstacles further increase the likelihood of slipping. Individuals with preexisting gait difficulty such as from back pain, Parkinson disease, multiple sclerosis, or stroke particularly slip easily.

Fainting (Fig. 1c) is due to impaired cerebral perfusion and transient brain hypoxia^51^, leading to a loss of postural tone. It is characterized by direct descent of the head and torso, while the CG remains in line with the feet, followed by bending of the torso and the knee, and then the whole body stumbles and collapses. Any pathology impeding adequately oxygenated blood flow to the brain can result in fainting, and this could range from chronic anemia, vasovagal syncope, paroxysmal arrhythmia, to dysautonomia, to name just a few.

Other common types of falls present as variations of stumbling or slipping. For example, falls develop while getting up from a chair (Fig. 1d) or sitting into it are commonly observed when the elderly use lightweight chairs or stools with wheels. Other examples include falling off or jumping down from a high structure such as ladders and desks (Fig. 1e,f), and tumbling down or slipping while walking down stairs, etc.

## Results

Our fall detection system conveniently comprises a computer, a camera, and a network system. The fall detection method is based on AI algorithms offered by SpeedyAI, Inc. The human detector achieves a held-out accuracy of 89% and a training accuracy of 94%. Please see the last paragraph in Detailed Methodology in the section of Methods for technical details.

**Figure 2:**
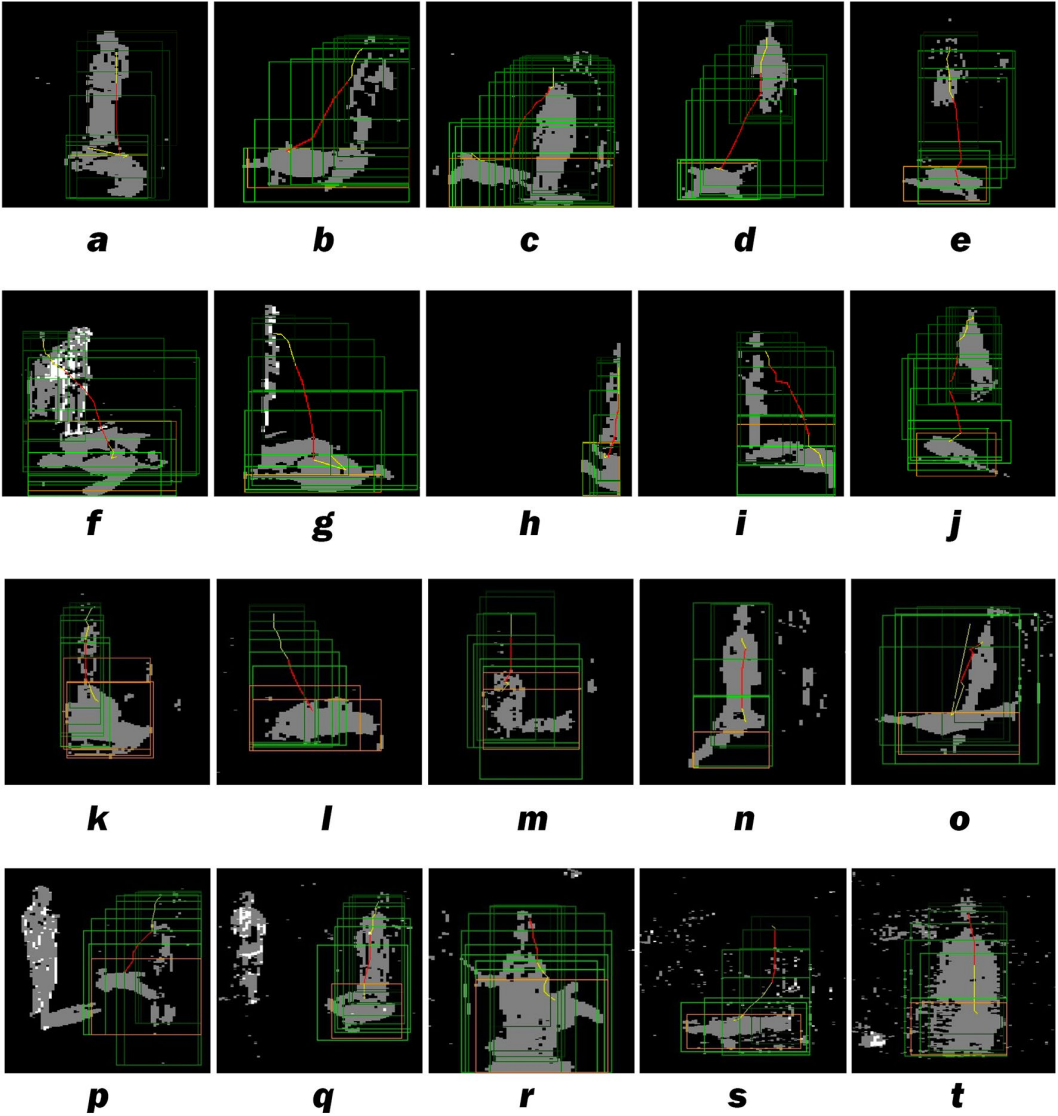
Our detection system used two very low-resolution cameras (so the system could process at high frame rates) to analyze fall patterns. Selected fall types under visible or infrared light captured by the system are displayed here. (**a**) Forward stumbling. (**b**) Sideway stumbling. (**c**) Backward stumbling. (**d**) Jumping down from a desk and falling. (**e**) Falling off a ladder. (**f**) Collapsing upon standing (i.e., Orthostasis). (**g**) Falling behind furniture (This situation is similar to a fall at the periphery of the camera viewing angle on the left). (**h**) Falling along the right edge of the viewing angle of the camera. (**i**) Falling partially out of the viewing angle of the camera. (**j**) Tumbling down stairs. (**k**) Forward fainting. (**l**) Sideway fainting. (**m**) Backward fainting. (**n**) Forward slipping. (**o**) Sideway slipping. (**p**) Backward stumbling in the presence of other people walking in the room. (**q**) Forward stumbling in the presence of other people walking in the room. (**r**) Forward stumbling with camera downward viewing angle greater than 45°. We crafted a rule to evaluate the inactive time. If the faller stays still for more than 10 s, the system will send the fall alarm. (**s**) Sideway stumbling behind a glass window covered by opened mini-blinds. (**t**) Forward stumbling behind a glass window covered by opened mini-blinds. Some of the short horizontal black lines in the figure represent the mini-blinds.

Selected fall types were evaluated (Fig. 2). Clockwise from the aerial view, Forward was defined as any fall occurring at 0° ± 45° with respect to the camera. Sideway was defined as any fall occurring at 90° ± 45° to the right and any fall occurring at 270° ± 45° to the left. Backward was defined as any fall occurring at 180° ± 45°.

**Figure 3:**
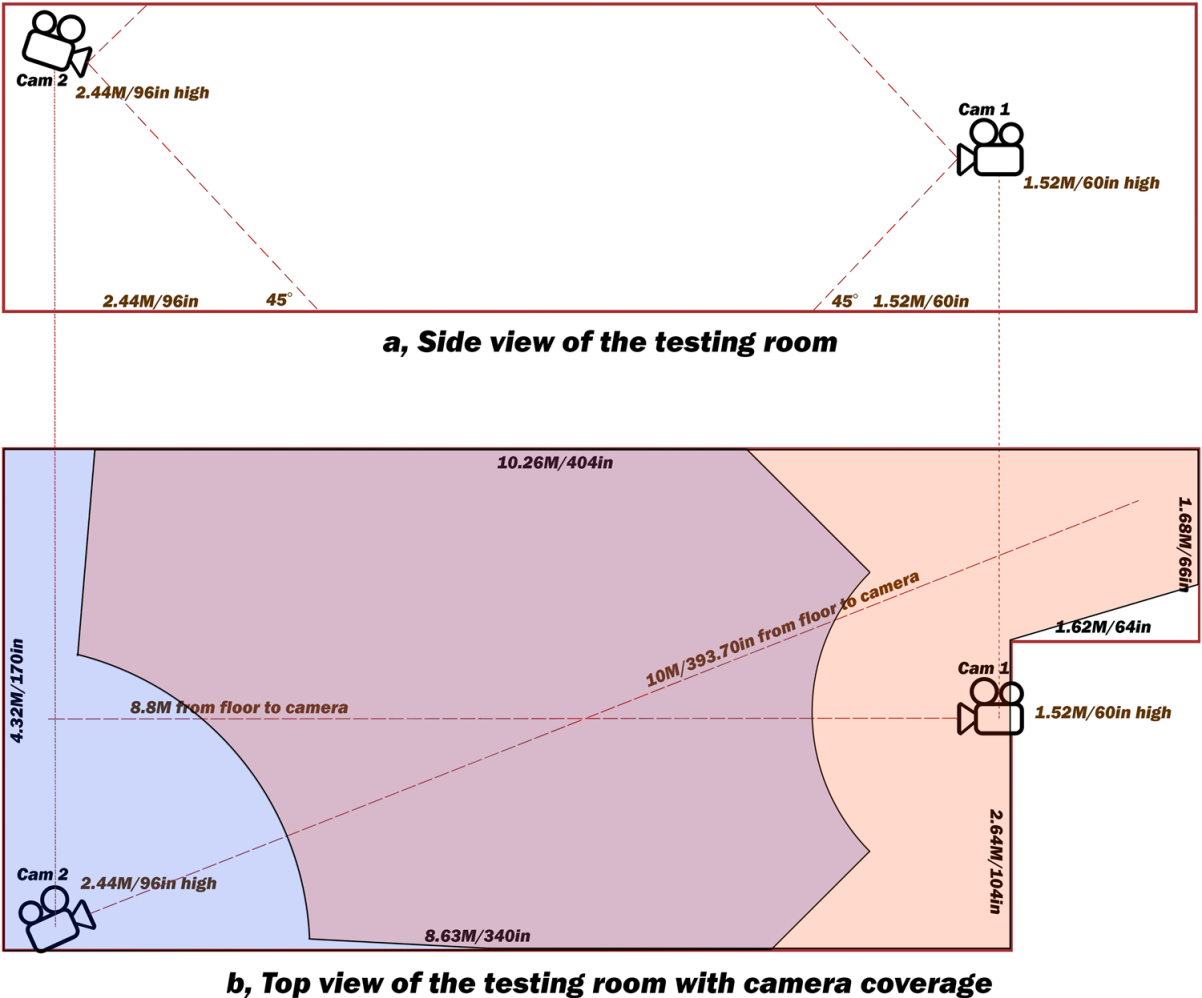
Overview of the testing room geometry and cameras’ locations. This figure demonstrates a two-camera setup that could catch any fall in any place inside the room (supported by our test set). (**a**) Side view. The lower boundary for fall detection was set to be aligned to the lower edge of the camera viewable area to maximize the widest detection. Complicated calibration was not necessary. (**b**) Orientation of the cameras for the testing site and their monitoring areas. The dotted lines extending from the camera lenses indicate where the fall tests were performed. Orange on the right is the inactive zone for Cam 1. Blue on the left is the inactive zone for Cam 2. Purple in the middle is the zone where falls are detected by both cameras. Precision and recall are increased in the purple zone. The presence of two cameras with this setup allows falls in any location in this room to be detected.

To evaluate the accuracy of our system, we tested two different Internet Protocol Cameras (IP Cam) installed at different locations as shown in Fig. 3. Camera one (Cam 1) was installed at 1.5 m above the ground and Camera two (Cam 2) at 2.5 m above the ground.

The first type of fall, Stumbling, was evaluated at distances of 2 m, 5 m, and 8 m from Cam 1, and 3 m, 6.5 m, and 10 m from Cam 2. The directions of the Stumbling were Forward, Sideway, and Backward. The number of Stumbling detected is listed in Table 1a. Please see falls detected and reported through Telegram Messenger in Supplementary Information, Section “Testing Data Figure a-w”.

**Table 1:** Detection results (Number of Falls Detected/Total Performed) of different types of falls and daily activities.

1a Detection results of Stumbling test via Camera One (Cam 1) and Camera Two (Cam 2). Fall detection accuracy via two cameras installed at different heights (1.5 m and 2.5 m) and measured at near, mid, and far distances. Test results were reported through Telegram messenger and attached in Supplementary Information, Section “Testing Data Figure a-w”						
**Distance from Cam 1**	**Forward** (number of falls detected/total performed)		**Sideway** (number of falls detected/total performed)		**Backward** (number of falls detected/total performed)	
2 m	N/A*		10/10		10/10	
5 m	10/10		10/10		10/10	
8 m	10/10		10/10		N/A**	
**Distance from Cam 2**	**Forward**		**Sideway**		**Backward**	
3 m	N/A*		10/10		10/10	
6.5 m	10/10		10/10		10/10	
10 m	10/10		N/A**		N/A**	

1b, Detection results of different types of falls via Cam 1						
Direction	Slipping	Fainting	Collapsing upon standing	Falling off a ladder	Jumping down from a desk and falling	
Forward	2/2	2/2	5/5	5/5	5/5	
Sideway	6/6	4/4	5/5	5/5	5/5	
Backward	2/2	2/2	N/A	N/A	N/A	

1c, Testing for false positivity on selected daily activities						
Distance from Cam 1	Bowing/Bending to 90°	Tying shoelaces	Push-ups	Sit-ups	Getting down to the floor	Jumping
2 m	0/30	0/30	0/30	0/30	0/30	0/30
5 m	0/30	0/30	0/30	0/30	0/30	0/30
8 m	0/30	0/30	0/30	0/30	0/30	0/30
**Distance from Cam 2**	**Bowing/Bending to 90°**	**Tying shoelaces**	**Push-ups**	**Sit-ups**	**Getting down to the floor**	**Jumping**
3 m	0/30	0/30***	0/30	0/30	0/30	0/30
6.5 m	0/30	0/30	0/30	0/30	0/30	0/30
10 m	0/30	0/30	0/30	0/30	0/30	0/30

*The lower border of the camera formed a 45° angle with the wall on which it was mounted. Installed at an arbitrary height X from the ground, the camera was expected to monitor falls taking place at a horizontal distance of X and beyond. An electronic fence along the lower edge of the viewable area of the camera was built to maximize the detectable area. Any fall hitting the fence would not get detected (e.g., a Forward fall). A second camera in the same room could be used to cover the inactive zone.

**Due to the room geometry, Backward and Sideway falls at the long distances would hit the walls and so were not performed.

***Crafted rules were applied (Please see “Discussion”) to distinguish true falls from the activity of Tying shoelaces at a  steep downward angle at near distances.

For the following five types of falls (Slipping, Fainting, Falling off a ladder, Jumping down from a desk and falling, and Collapsing upon standing as in orthostasis), the number of falls detected is listed in Table 1b. Cam 1 was again installed at 1.5 m above the ground. In particular, Falling off a ladder and Jumping down from a desk and falling were monitored horizontally 6 m away to accommodate the extra height. The other types of falls were detected 5 m horizontally away from Cam 1 (Table 1b).

Daily non-fall activities such as Bowing/Bending to 90°, Tying shoelaces, Pushups, Sit-ups, Getting down to the floor, and Jumping were tested in all directions (ten times for Forward, five times for Sideway to the left and five times for Sideway to the right, and ten times for Backward) for a total of 30 times by both Cam 1 and Cam 2, each at near, mid, and far distances (Table 1c).

**Figure 4:**
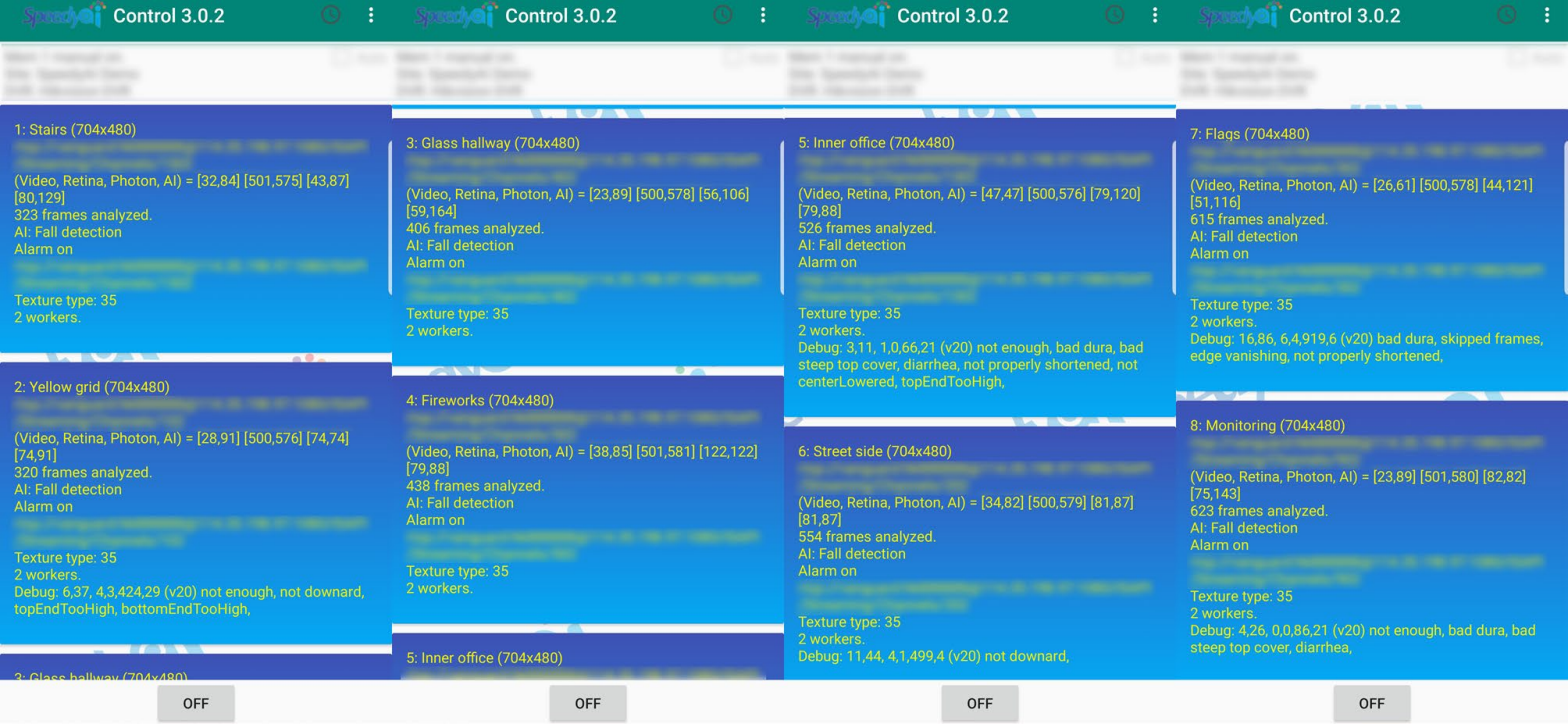
Four screenshots of the control panel demonstrating eight cameras monitored simultaneously by an eight-core Android TV box. The AI time (the last pair of the four sets of numbers, or the second row after the first redacted lines) indicates the minimum and maximum time in milliseconds during which the system processes a video frame. The average frame rate was around 10 FPS, meeting our detection requirement. This eight-core Amlogic S912 CPU loading during the monitoring period was between 43 and 85%. No falls happened, were detected, or reported during the testing period. The video signal was sent through internet connection. Some of the information in this figure was redacted for privacy as the videos in this surveillance system were obtained to monitor a company’s office and its vicinity.

Finally, a 16-channel Digital Video Recorder (DVR) system was used to test the performance of our system. A quad-core Cortex-A53 device (programmed from a low-cost (~ $30) conventional Android box), an octa-core Cortex-A53 device (programmed from a medium-priced (~ $40) conventional Android box), and a quad-core Cortex-A72 device (Linux-run Raspberry Pi 4 (~ $45)) were all tested for maximum camera connections. A video stream at at least 10 FPS was required for accurate fall detection (See details in “Discussion”. The result demonstrated that the quad-core Cortex-A53 system could simultaneously monitor 6 cameras; the octa-core Cortex-A53 system could simultaneously monitor 8 cameras (Fig. 4); and the quad-core Cortex-A72 Raspberry Pi 4 could simultaneously monitor up to 12 cameras and continuously for 4 weeks without overheating the device. Specifically, an octa-core Cortex-A53 device has been running for over 5 months for the stress test, failed only twice due to power outage, and as of this writing is still running. Note that simultaneous monitoring refers to reliable detection even when human falls simultaneously happen in all the monitored cameras at any given time. This is in contrast to many current multi-camera AI systems, which, due to Central Processing Unit (CPU) overload, work only if simultaneous triggering events happen in only a few (2 or 3) of the monitored cameras at any given time.

**Figure 5:**
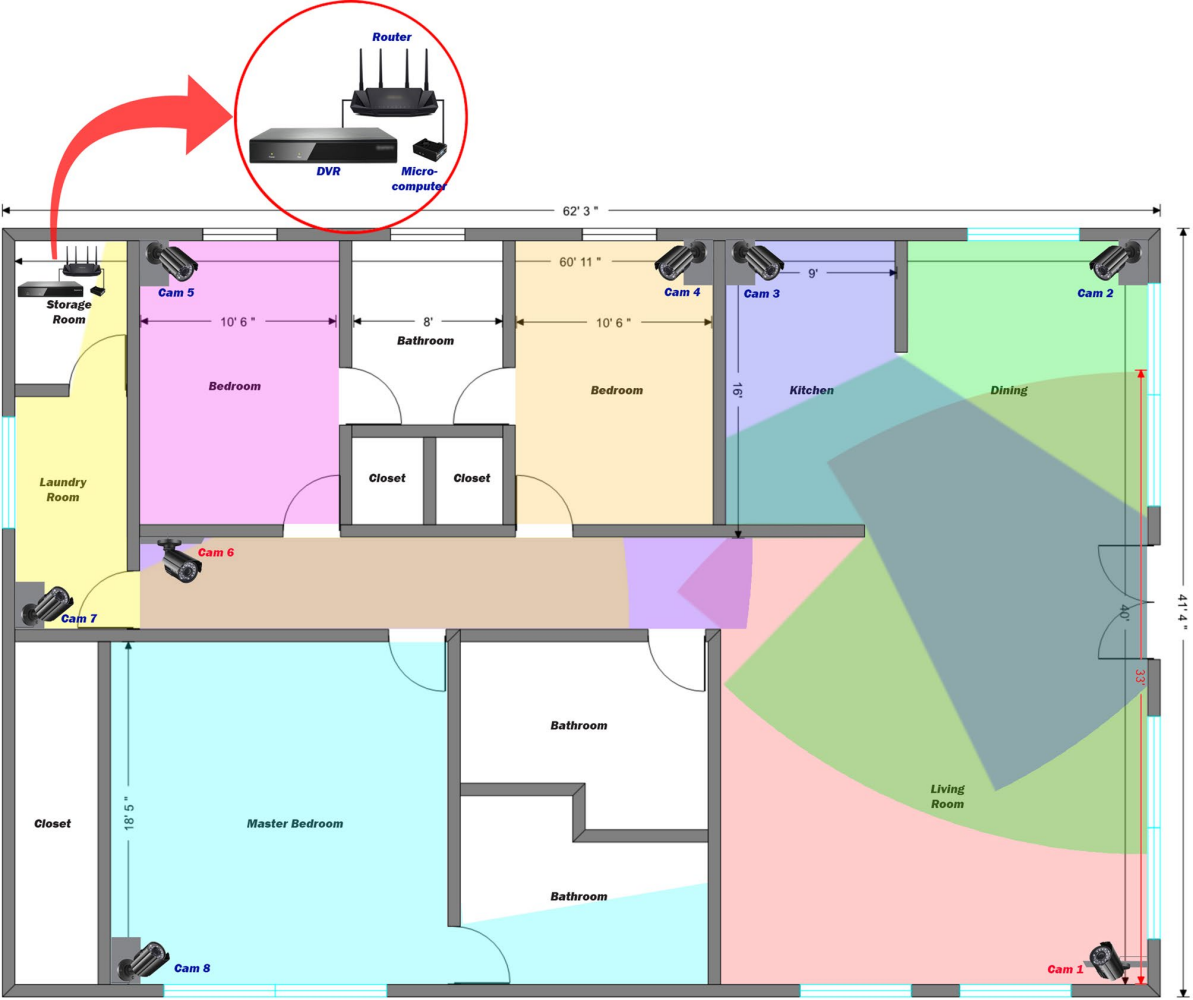
An example of an eight-camera installation system in a three-bedroom house. The size of the house is approximately 2400 ft^2^. Two cameras were installed at 2.5 m high to avoid occlusion by tall furniture (Cam 1 & Cam 6). The other six cameras were installed at 1.5 m to ensure the best detection possible. The installed cameras detect falls at 640 by 360 pixel resolution, which was down-sampled from the original resolution to allow our CPU to handle eight cameras, with a 110 degree view angle diagonally. The horizontal view angle is 110° × [704/(7042 + 4802)0.5] = 91°, and the vertical view angle is 110° × [480/(7042 + 4802)0.5] = 62°, where 704 depicts the camera’s horizontal resolution and 480 depicts the camera’s vertical resolution in pixel. The maximum distance that the system can cover is 10 meters (33 ft). Many of the areas were monitored by two or three cameras.

Figure 5 illustrates an example of the installation of the whole system in an average American house.

## Discussion

We proposed a novel fall monitoring system by analyzing the behaviors of the apparent human fall heights, velocities, and accelerations utilizing speedy AI algorithms, IP cameras, and an affordable microcomputer. Similar to previous works, our system uses single cameras to detect falls. However, unlike previous works, our highly efficient inference algorithms enabled the concurrent processing of a vast amount of data captured from as many as eight cameras using a low-cost CPU such as Amlogic S912 at ten or higher FPS. Consequently, multiple cameras installed at different locations to monitor the same area provide a higher accuracy than single cameras could. Alternatively, if multiple cameras are installed in different rooms, a house-wide coverage can be offered. Our approach of analyzing the human fall pattern enabled the use of low-resolution cameras while simultaneously achieving high detection precision and recall. If moderate or high-resolution cameras are used, the system functions equally efficiently, allowing much longer distance monitoring in long hallways of 60 m and beyond (Please see calculation details in the fourth to the last paragraph in “Discussion”). In addition, the system can differentiate true falls from regular activities, capture falls partially occluded by furniture, and detect falls through glass and water, and in the presence of other moving people or animals in complex backgrounds. Finally, our model allows connection to an already-existing surveillance system, encompassing the use of downward-viewing cameras, for dual purposes of security and fall detection, as many households and senior centers already have such an installation. Our vision is that the highly accurate, efficient, and affordable fall detection system will minimize as much fall-related disability and fatality as possible. Our mission is to mass implement the system to ensure safety in senior homes, rehab centers, nursing facilities, and high-risk zones.

Please see the comparisons of performance and cost of our system with the previously published works (Systems A and B) and current commercial products (Systems C and D) in Table 2.

**Table 2:** Comparisons of performance and cost of our system with the previously published works or current commercial products.

	Our System	System A	System B	System C	System D
Detection Method	Single camera	3D camera	Single camera	Single camera	Single camera
Resolution (pixels x pixels)	640 × 360 or higher	Depth: 1280 × 720 RGB: 1920 × 1080	320 × 240	Unknown (Information not available publicly)	Unknown (Information not available publicly)
Frame Rate (FPS)	At least 12 (processed)	Depth: 90 RGB: 30 (Only camera frame rate reported)	7–8 (processed)	Unknown (Information not available publicly)	Unknown (Information not available publicly)
Detectable Distance (m)	10 (foreground)	3.5–6 (foreground) 10 (background)	10 (foreground)	Unknown (Information not available publicly)	Unknown (Information not available publicly)
Single or multiple targets per video	Multiple, non-overlapping	Single	Single	Single	Single
Hardware	Android TV Box Eight ARM Cortex-A53 cores, 2 GB RAM	Intel Movidius per camera	Rasberry Pi 2 Four ARM Cortex-A53 cores	Hardware sensor + server computers	Hardware sensor + server computers
Can detect through transparent glass or screen	Yes	No	Yes	Unknown (Information not available publicly)	Unknown (Information not available publicly)
Rooms/Areas Monitored	8	1	1	6	6
Estimated Cost ($)	8 cameras with a recorder: $150-$300 online 1 Computer for 8 cameras: ~ $40 Total estimated cost per room: ~ $24 to $43	Camera: $199 Video Processing Unit: $70 Computer: ~ $500 Total estimated cost for one room: ~ $750	Camera: $50 1 Computer for 1 camera: ~ $40 Total estimated cost per room: ~ $90	Two sensors for two rooms + server computer: $1,600 Maximum 6 room: $2,000 Monthly charge per camera: $30 Total estimated cost per room: ~ $333 + $30/month *As of March 2019*	Camera: $500 (Installed) Monthly charge per camera: $100 Total estimated cost per room: ~ $500 + $100/month *As of March 2019*

To design a practical and affordable house-wide fall detection system with high accuracy and capability of multi-video monitoring, it was crucial to have a high-speed AI algorithm without a dedicated AI processor. However, even in the presence of a fast AI system, we still needed to further reduce the processing load, so background subtraction to reduce the pixel depth from 24-bit to 1-bit to analyze the fall pattern was necessary. The added benefit was the ability to detect falls at a much longer distance, but with the consequence of having less information which might compromise the AI prediction. To overcome this barrier, we would need to craft some rules to boost the accuracy in scenarios when the detection was not optimal. Finally, it was logical to use the widely available, inexpensive surveillance system which might have been already installed, for both home security and fall detection purposes. In this case, our AI needed to be trained to identify falls from downward-viewing angles besides from horizontal positions as seen in previously published studies.

**Figure 6:**
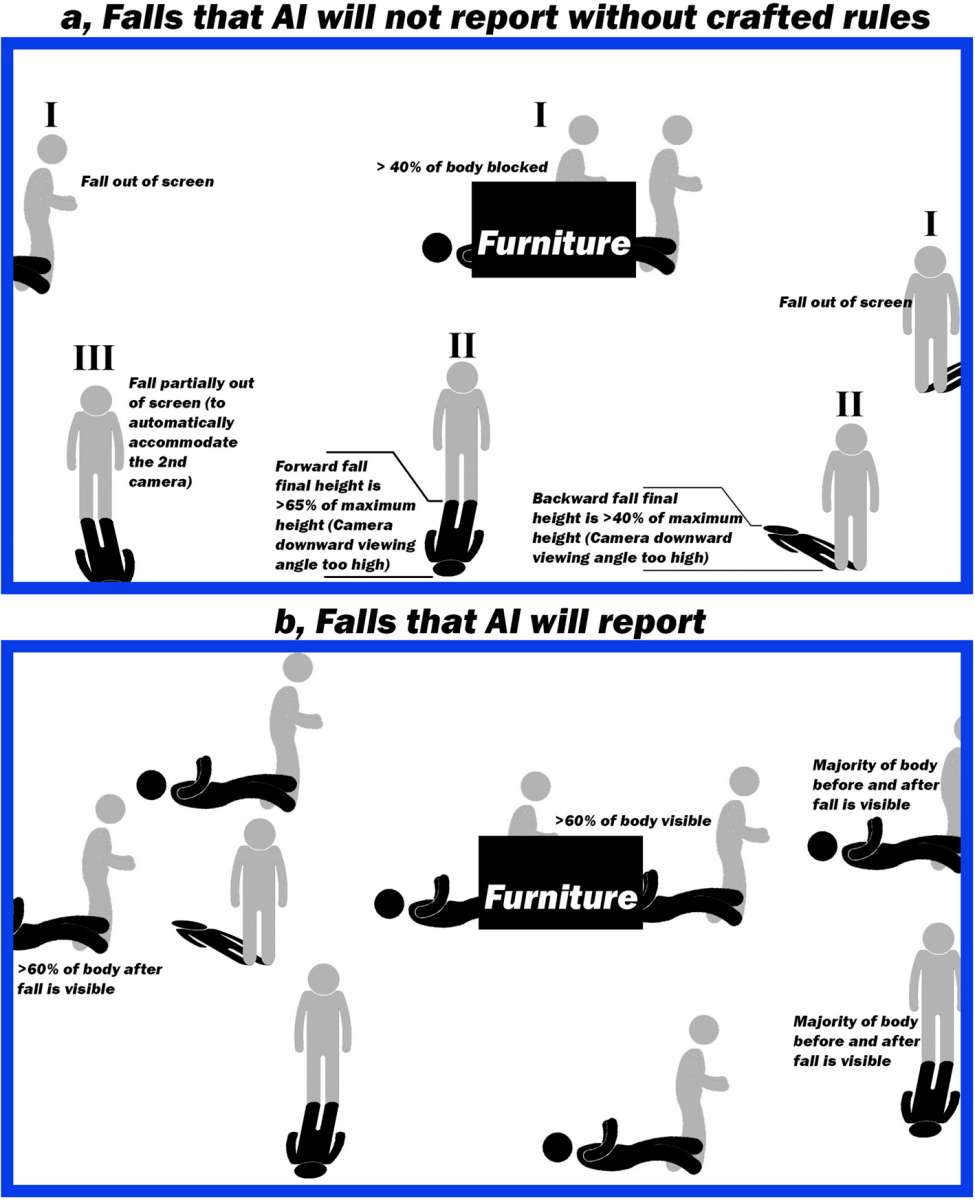
Predefined scenarios of falls the AI will not report without crafted rules and selected examples of falls the system will report. (**a**) Scenario I: When a person or > 40% of a person falls off of the screen, the fall will not be detected, because there is not enough body in the camera view for detection. Scenario II: If the downward viewing angle is greater than 45°, the apparent height after a fall will be longer than 40% of the maximum height during the backward fall, and longer than 65% of the maximum height during the forward fall. These scenarios indicate that the camera angle is not properly adjusted; in this case, we crafted some rules to extend the detectability of the falls. To further enhance detection at steep downward angles which may otherwise have poor accuracy, we measure the fall pattern and inactive time. These crafted rules allow forward fall detection with apparent final heights extending from 65 to 95%, and backward fall detection with apparent final heights extending from 40 to 70%, at steep downward camera viewing angles. Scenario III: When a person touches the predefined boundary (also known as the electronic fence), a part of the fall pattern may not be properly analyzed. The system will need to use the crafted rule of inactive time to maximize detection efficiency. A second camera can also be used at a different location to optimize the coverage and improve the detectablility as demonstrated in Fig. 3. No additional calibration is needed. More crafted rules are explained in “Methods”. (**b**) Selected examples of falls the system reports. When > 60% of the body is visible on the screen and does not touch the predefined boundary (lower edge of the screen) after the fall, it will be reported.

Our model was capable of detecting various types of falls: Stumbling, Slipping, Fainting, Collapsing upon standing (orthostasis), Falling off a ladder or any high structure, and Jumping down from a high altitude and falling (Table 1). Despite the near 100% accuracy, there were few false-positives on the activity of tying shoelaces initially. One reason was because the actor bent down very quickly (< 0.3 s) and the system mistook this action as a free fall. Secondly, this action occurred near 45° angle (lower edge of the camera view), precluding a proper analysis. To circumvent these issues, we have crafted a few rules. First, an electronic fence along the lower edge of the camera was built to maximize the detectable area. Any fall hitting the fence would not be reported by the AI unless passing our crafted rules (Fig. 6a). On the other hand, any fall beyond the fence could be correctly detected without any complicated calibration when the lower edge of the camera was set at 45° (Fig. 6b).

Other crafted rules further enhanced the differentiation of true falls from many daily activities, such as running, sitting, standing, bending forward, doing push-up, and leaning again a wall, and all could be monitored by either horizontally or high altitude-mounted cameras (Fig. 2r). Using a low-resolution camera (e.g., 640 pixels by 386 pixels with 110° viewing angle), the AI with crafted rules could identify falls up to ten meters away. This method was not affected by transparent glass or plastic doors, opened mini-blinds, showers, rain, etc. (Fig. 2s,t). In addition, the AI system could track multiple people in the room, isolate the faller from others in motion (as long as they do not overlap from the camera’s point of view), and detect falls between furniture.

A purposeful jump followed by tumbling as seen performed by a gymnast may also present with a high velocity and acceleration close to the true fall, and mistakenly be detected as a fall and triggers the alarm. The crafted rules can analyze the post-event action and accurately distinguish a purposeful non-fall event from a true fall.

A fall is an uncontrolled action in which one acutely collapses in a short time. From the training dataset, we concluded that a frame rate of 7 FPS was necessary to produce a result close to 90% recall and greater than 90% precision. Considering the limited GPU bandwidth, we were able to set our system to a higher frame rate at 10 FPS to ensure sufficiently effective data whilst using as many as eight to twelve cameras.

For distance capability, our detection method accurately detected falls at 10 m, with Cam 1 configured at a low resolution of 640 by 386 pixels, and Cam 2 configured at 640 by 354 pixels. The detectable distance could easily reach beyond 30 m using a camera with 1920 by 1080 resolution (10 m × 1080 pixels/354 pixels = 30.5 m). Similarly, the detectability could reach beyond 60 m using a 4 K-resolution camera (10 m × 2160 pixels/354 pixels = 61.0 m), provided that the computer-camera connection is capable of feeding at least a 10-FPS video, with the same viewing angle (110° diagonal). In this case, a more powerful single-board computer such as Raspberry Pi 4 would be able to handle such a task. In fact, the AI fall detection algorithm was very efficient that the computation bottleneck was usually in the video feed instead of the AI.

The location of the camera is important, and the best measurement angle is parallel to the ground and at low camera heights. However, low camera heights make detection difficult when there are occlusions such as chairs and sofas. Nevertheless, the AI technology could overcome this obstacle as long as at least 60% of the human body was not occluded by furniture. In addition, elevating the camera to up to 45° could also minimize occlusion. Specifically, Cam 2 installed at 2.5 m high detected all types of falls in our test. Moreover, in a complex environment with falls occurring at all different angles and complicated by the presence of furniture, installation of multiple cameras at different locations and heights would enhance the detection rate. The system used in the experiment could monitor eight cameras simultaneously for fall detection.

This fall monitoring system requires very little computer memory (less than 50 kilobytes per camera) given that the involved computation is very lightweight, and the program can be installed onto a cost-effective minicomputer like Raspberry Pi or Android television box, which receives video feeds from IP Cams or surveillance DVR, without the need of a high-end Neural Processing Unit (NPU) and Graphics Processing Unit (GPU). One or more affordable cameras can be installed in every room, so the whole house can be monitored at a reasonable cost. Since all the videos are processed locally, privacy is preserved. Once a true fall is detected, the alarm can notify families, neighbors, security guards, or paramedics through messaging services such as Phone Messages, WhatsApp, Telegram, Line, or WeChat. This decreases the  manual monitoring burden on family members and social workers.

In the future, several avenues can be taken to enhance this fall detection system. Currently, the quantity and variety of our fall data are limited because generating fall videos can create injuries, thus many of the falls were produced by a dummy. It is more practical to obtain a larger amount of real-life data from rehab centers, senior homes, and medical facilities to ensure statistical robustness. The pediatric population could also be included. The AI algorithms could be refined to separate shadows from humans and the performance should be improved. Ultimately, fall prevention will be the next necessary step to advance care.

## Outlook

We have developed a low-cost, multi-camera, house-wide fall detection system with high accuracy. Its fall detection capability at long distances, through transparent objects, behind furniture, and in the presence of other people or animals in the same room demonstrates its practicality and feasibility in real life. The system’s flexible installation further highlights its potential for public use in the future. By addressing the global problem of falls at an early stage to minimize any subsequent disability and fatality, we hope our research has the potential of improving the current clinical practice of fall detection and creating a paradigm for future studies in fall detection technology.

## Methods

All methods were carried out in accordance with guidelines and regulations of UCLA HIPAA, Good Clinical Practice, Biomedical Research, and FDA Regulated Research. The training and validation procedure carried out conforms to the standard machine learning protocol [Bishop, C. Pattern Recognition and Machine Learning (Springer, 2006)]. No human research subjects were recruited. The image and video data used in this research work were obtained from already-existing, publically available data from the internet (for training and validation only) and the actual testing videos were generated and performed by the authors using a dummy model.

We propose a novel concept of measuring velocities and accelerations for fall analysis. To our knowledge, currently all image-based algorithms monitor falls by calculating the tilt angle between the faller and ground, but this approach fails when the fall is not perpendicular to the optical axis. Although the 3D depth meter addresses this issue, it cannot recognize humans partially blocked by obstacles or behind transparent objects such as glass windows. To solve these problems, we decided to measure the velocities and accelerations that comprised the fall.

### Critical parameters for fall analysis

**Figure 7:**
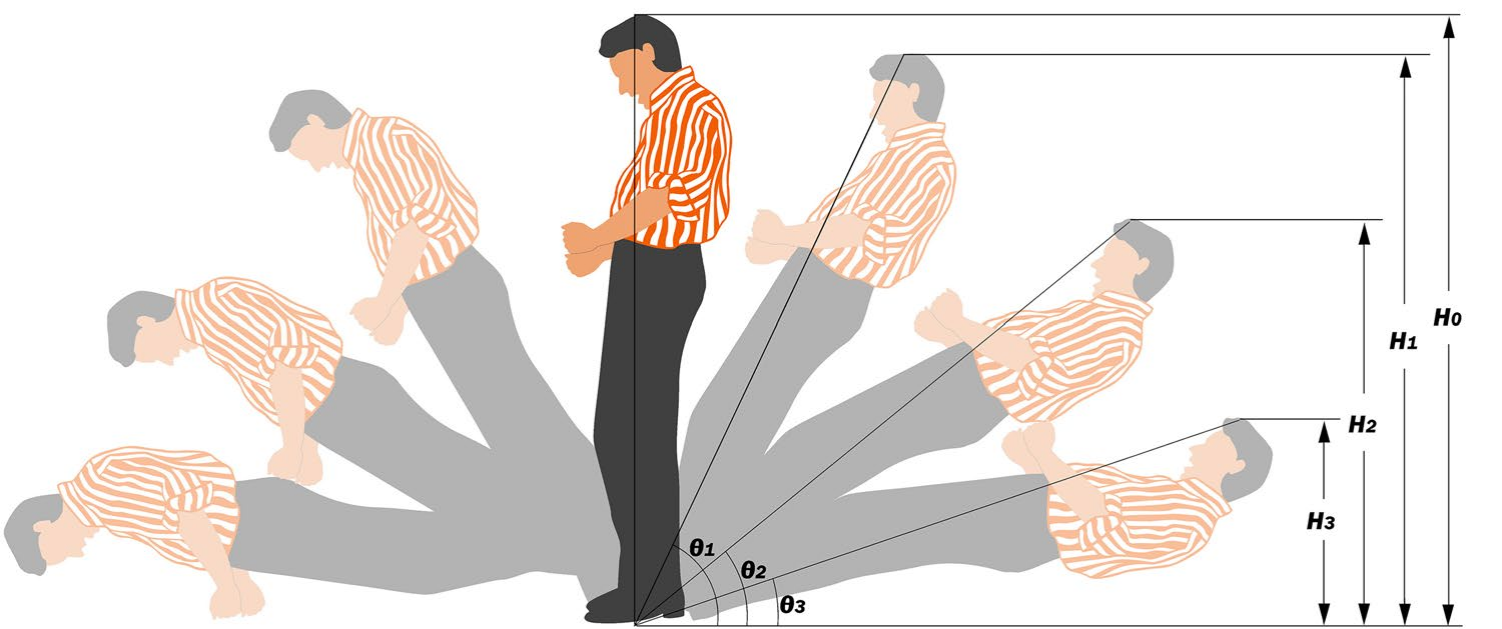
Change of apparent heights and tilt angles during a fall.

The current most commonly used threshold to determine a fall is the tilt angle *θ*_*i*_ (Fig. 7), and it can be obtained by the following equation:

$$\frac{{H_i}}{{H_0}}$$

where *H*_*0*_ is the original height, or total body length, of the person before the fall, and *H*_*i*_ is the apparent height measured vertically from the ground to the vertex of the head when falling at the tilt angle *θ*_*i*_. This ratio of ${\text{H}}_{\text{i}}/{\text{H}}_{0}$ can be obtained even if the direction of the fall is along the optical axis.

In real life, however, a person’s posture hardly remains straight during a fall, and so a fixed value of *θ*_*i*_, may not allow the recognition of a true fall. Also, this approach may mistake an exercise such as a pushup or sit-up, or a position such as leaning against a wall, as a fall, if only based on a fixed *θ*_*i*_.

In order to solve these dilemmas, we propose a novel concept of using velocities and accelerations to detect a fall by first calculating the difference in the apparent heights between the (*i-1*)th and *i*th camera frames:

1 $${{V}}_{{i}}{=}\frac{\left({{H}}_{{i-1 }}-{{H}}_{{i}}\right)}{{{H}}_{0} \, {\times}{ (}{{T}}_{{i}}{- }{{T}}_{{i-1}}{)}}$$

where *T*_*i*_ and *T*_*i-1*_ are the times at *H*_*i*_ and *H*_*i-1*_, respectively. *H*_*0*_ is used to normalize the distances between the camera and the faller.

The acceleration equation is expressed as follows:

2 $${{a}}_{{i}}{=}\frac{{{V}}_{{i}}-{{V}}_{{i-1}}}{\left(\frac{{{T}}_{{i}}-{{T}}_{{i-2}}}{2}\right)}{=}\frac{\left(\frac{\left({{H}}_{{i-1 }}-{{H}}_{{i}}\right)}{{{H}}_{0} \, {\times}{ (}{{T}}_{{i}}{- }{{T}}_{{i-1}}{)}}-\frac{\left({{H}}_{{i-2 }}-{{H}}_{{i-1}}\right)}{{{H}}_{0} \, {\times}{ (}{{T}}_{{i-1}}{- }{{T}}_{{i-2}}{)}}\right)}{\left(\frac{{{T}}_{{i}}-{{T}}_{{i-2}}}{2}\right)}{=}\frac{2}{{{H}}_{0}}{\times}\left(\frac{\left({{H}}_{{i-1 }}-{{H}}_{{i}}\right)}{{(}{{T}}_{{i}}{- }{{T}}_{{i-1}}{)}{\times}\left({{T}}_{{i}}-{{T}}_{{i-2}}\right)}-\frac{\left({{H}}_{{i-2 }}-{{H}}_{{i-1}}\right)}{{ (}{{T}}_{{i-1}}{- }{{T}}_{{i-2}}{)}{\times}\left({{T}}_{{i}}-{{T}}_{{i-2}}\right)}\right)$$

In a video stream, the time between each frame is constant (1/29.97 s in National Television System Committee (NTSC) standard). If the detection model is faster than the video, then the equation can be further simplified into below:

3 $${{a}}_{{i}}{=}\frac{\left({{H}}_{{i-1 }}-{{H}}_{{i}}\right)-\left({{H}}_{{i-2 }}-{{H}}_{{i-1}}\right)}{\Delta {{T}}^{2}{\times}{{H}}_{0}}{=}\frac{{{2H}}_{{i-1 }}-\left({{H}}_{{i}}{+}{{H}}_{{i-2 }}\right)}{\Delta {{T}}^{2}{\times}{{H}}_{0}}$$

In the actual computer program, we used the numerical approach, and all the values we were looking for here were relatively easy to calculate. Now we would have several values: height (*H*_*i*_), width (*W*_*i*_, calculated from height and angle), tilt angle (*θ*_*i*_), velocity (*V*_*i*_), acceleration (${\text{a}}_{\text{i}}$), and time between frames ($\Delta \text{T}$). All the videos were preprocessed by removing the background (background subtraction), and the silhouette video was used to extract these parameters for the AI algorithm to analyze.

### Dataset collection

A 1080P30 High Definition (HD) camcorder was used to record the training data. Each video included four sections: 1. A background section of more than 60 s used to subtract the non-moving objects from the current frame; 2. An initial-fall phase during which a person began to descend and reach an angle at which one could not control one’s torso. We initially defined the angle change as from upright of 90° to 60° angle. This angle was further optimized as the AI training proceeded; 3. A true-fall phase during which one can no longer prevent the fall. We initially set the angle from 60° to 0° (on the ground); and 4. A post-fall phase for a few seconds. Instead of feeding the whole video to an AI model, we only fed the four tagged sections with following six parameters: height (${\text{H}}_{\text{i}}$), width (${\text{W}}_{\text{i}}$), tilt angle (${\theta}_{\text{i}}$), velocity (${\text{V}}_{\text{i}}$), acceleration (${\text{a}}_{\text{i}}$), and time between frames ($\Delta \text{T}$), for the AI algorithm to learn the pattern.

The video was then sub-sampled to different frame rates to compare the recall and precision. We determined that, in our model, a minimum of 7 FPS was necessary to produce a result close to 100% recall and greater than 90% precision, so we settled the frame rate at 10 FPS for our system to analyze. Base on the GPU bandwidth, we then chose the camera resolution to meet our need of at least eight cameras at 10 FPS each.

The video was collected on all different types of fall. Six types were included in the dataset to train our system. The Stumbling video was produced using a dummy with a string tied around the head and pulled from different angles with 30° increments (from the top view) to produce the fall. The Slipping video was produced using a dummy with a string tied to the leg and pulled from different angles with 30° increments to produce the fall. The Falling off a ladder video was produced similar to the Stumbling video with the dummy placed on an elevated platform (e.g., desk) and a string tied around the head. The Jumping down from a desk and falling video also used the dummy with a string tied around the waist. For Fainting and Collapsing upon standing videos, the falls were performed by a real human to generate the video training set.

Stumbling, Slipping, Falling off a ladder, Jumping down from a desk, Fainting, and Collapsing upon standing were repeated 5 times on each fall angle. Collapsing upon standing, Falling off a ladder, and Jumping down from a desk and falling were performed at angles 0°, 30°, 60°, 300°, 330°, and the rest fall types were performed at all angles 0°, 30°, 60°, 90°, 120°, 150°, 180°, 210°, 240°, 270°, 300°, and 330°.

For non-fall video collection, Bowing/Bending, Tying shoelaces, Push-ups, Sit-ups, Getting down to the floor, and Jumping were all collected using a real person, five videos for each angle (0°, 30°, 60°, 90°, 120°, 150°, 180°, 210°, 240°, 270°, 300°, and 330°). All videos consisted of a one-minute background (standing still) section followed by one action section. Most of the non-fall dataset was generated in-house. A few other non-fall data was added to balance the weighting of fall and non-fall data during the training. We entered keywords such as “sports video”, “walking video”, and “jogging video” on Google and obtained a few clips. Since these data are non-essential and small in quantity, we collected randomly from internet/TV/Youtube.

All videos collected in-house used a DXG-5K1V camcorder in 1080P30 mode, zoomed to 10X to keep the viewing angle as close to horizontal as possible. This was especially important for the Falling off a ladder, and Jumping down from a desk and falling actions. Manual focus was selected to prevent any possible artifact caused by hunting (sudden change in focus).

### AI training

Successful fall detection requires successful human detection first. We surveyed several human detection models and selected the AI algorithm offered by SpeedyAI, Inc. because it was the most efficient for both computation and memory footprint. To further expedite the detection pipeline, we improved the SpeedyAI algorithm to only focus on the areas in motion to identify the moving humans in these areas (Fig. 1). The output would be all the moving humans’ locations and dimensions. This allowed a detection speed of more than 20 FPS on the 1080 progressive scan (1080p) video. We input a video dataset consisting of falls and daily activities into the human detection model we created. Then we fed these processed outputs to the fall detection model we crafted, using leave-one-out cross-validation (given the limited amount of data) to train and set the parameters accordingly. A second held-out dataset was used to evaluate the accuracy. Real-life fall detection in different directions and distances was then performed. Please see detailed methodology below.

### Detailed methodology

We collaborated with SpeedyAI, Inc. for a joint research effort to tackle the challenging problem of reliable human detection AI technology to achieve high-frame-rate detection in the context of high-resolution video scans. The critical drawbacks with the current state-of-the-art object detection algorithms based on deep learning and variants of Convolutional Neural Networks (CNN) are the high load in Central Processing Unit (CPU) cycles, memory footprint, and heat dissipation, despite recent improvements such as YOLO^52,53^. These drawbacks are even more pronounced given the fact that our fall detection paradigm exists in an edge device, whose computation resources are minimal. Indeed, for cost-effective large-scale deployment of our fall detection system to maximally benefit the people at risk of falls, it is paramount that we make it operate reliably in a low-cost hardware. To this end, we proposed an AI learning methodology that steered away from the now famous deep learning/neural network paradigm to what is perhaps one of the most underrated learning algorithms, the Relevance Vector Machine (RVM)^54^. Please see Supplementary Information, Section “Relevance Vector Machine (RVM) Learning Algorithm”, for more details.

Even before the recent resurgence in popularity of deep neural networks since 2012^55^, Support Vector Machine (SVM) was the de facto learning algorithm used in the machine learning and computer vision community. The formula of a learning system in the past was engineering one’s data features, feeding them to an SVM, and publishing one’s paper. This was so, despite the fact that for many learning tasks a better alternative to SVM existed. For example, unlike RVM, SVM is fundamentally a classifier that does not produce probabilities. It is therefore interesting that when probabilities were desired, people patched SVM into producing probabilities instead of using inherently probabilistic models. In fact, numerous researches were devoted entirely to this endeavor^56^.

For our fall detection technology, RVM is important for various reasons. First, it inherently produces probabilities, whose importance for our task will be discussed shortly. Second, it produces much sparser models than an SVM, which is essential in how we can make our computation fast enough for real-time application on a low-cost edge device. Third, RVM has fewer parameters to tune; this is especially important to guard against overfitting because our training dataset is not very large, as fall data are extremely costly to acquire. Fourth, unlike SVM, it can mathematically be formulated as a multi-class classifier, facilitating other researchers to extend our current work. On the other hand, one drawback RVM has is its much longer training time. Also, it is much less popular than SVM and has no well-maintained software packages available. We had to design and implement it from scratch.

For the human detection technology, we worked with SpeedyAI, Inc. with the permission to use its proprietary, manually labeled dataset of 50,000 human images in uncontrolled, real world settings. With an additional dataset of 100 K background images manually inspected not to contain any humans or human parts, we trained an RVM using the Histogram of Oriented Gradients^57^ (HOG) features extracted from each color channel (red, green, and blue) from each image. We chose HOG (Please see Supplementary Information, Section “Histogram of Oriented Gradients (HOG) Feature Extraction” for more details) as our input features because it could be computed quite quickly, without incurring too many CPU cycles. Our particular implementation further optimized HOG feature transformation by using various CPU and architecture-specific techniques.

**Figure 8:**
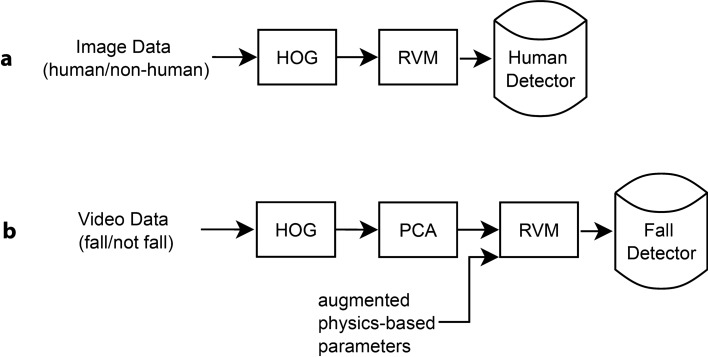
The main pipeline for training (**a**) our human detector and (**b**) our fall detector.

The main pipelines for training our human detector and fall detector are shown in Fig. 8a,b, respectively. Our version of the HOG feature engineering has some subtle uniqueness and is discussed in detail in the Supplementary material along with the RVM learning framework. As RVM works on completely arbitrary feature space, it is straightforward to introduce different features into learning in a unified manner. In particular, for our fall detector, adding hand-crafted features or physics-based parameter features (e.g. acceleration) in addition to image semantics-based features is simply a matter of adding more columns to the design matrix Φ. The final human detector trained had a payload size less than 20 KB.

With the fall video data collected as described in the previous section, training our fall detector was similar to training our human detector. However, several critical differences need to be emphasized. Instead of extracting features from the entire frame of pixel space where motion happens, we first cropped out the subregion in which the (possibly falling) human was present. We applied our feature transformation to this region, and discarded the rest of the frame. This sifting step encouraged the system to focus only on the most important visual content, the fall itself. In practice, one of the few major hurdles was that now we had temporal data in videos instead of static data like images as before. This hurdle prevented us to readily use the RVM we had developed, as each video consisted of much larger data payload on the number of pixels, in contrast to an image. This posed a serious problem classically known in the machine learning literature as “fat feature matrix”, in which the number of features far exceeded the number of training data instances. This was problematic in the same way that, with sufficiently many people encountered, someone's social security number would align perfectly with tomorrow's stock prices even though such spurious correlation would be entirely useless for generalization and stock price prediction.

To curb this issue, we applied standard techniques to reduce the dimensionality of our features. As cautioned in Hastie et al.^58^, dimensionality reduction was done before any involvement of the ground truth labels. First, as before, we applied HOG feature transformation to each frame in the video and concatenated them. Then, we used linear Principal Component Analysis (PCA) to make our data matrix leaner. In the end, we were able to reserve 95% of our training data variation whilst reducing the dimensionality of our features by more than 99%. As these features would no longer be histograms after PCA transformation, a histogram intersection kernel cannot be applied. Therefore, unlike the case with human detection, Φ lives in a linear feature space rather than a kernelized one. There were other more involved techniques for dimensionality reduction, such as kernel-based PCA or other kernel transformations. We left these choices and their implications as future studies for our work.

Armed with the extremely tiny yet accurate human image detector and human fall video classifier, we were ready for our final fall detection system. First, for each video frame feed, we applied the human detector as a sliding window of various sizes scanning across the frame, focusing on regions where background substraction indicates presence of motion. In other words, for each frame F, we obtained a list of subregions in F, say A_1_, B_1_, …, each of which had a probability a_1_, b_1_, … of having a human presence. Now, for a sequence of frames, we had frame-level sub-regions as follows:

Frame 1: A_1_, B_1_, C_1_, …

Frame 2: D_2_, E_2_, …

Frame 3: F_3_, G_3_, H_3_, I_3_, …

Frame 4: J_4_, K_4_, L_4_, M_4_, N_4_, …

Each of the above had a human-presence probability as computed by our human detector as follows.

Frame 1: a_1_, b_1_, c_1_, …

Frame 2: d_2_, e_2_, …

Frame 3: f_3_, g_3_, h_3_, i_3_, …

Frame 4: j_3_, k_4_, l_4_, m_4_, n_4_, …

**Figure 9:**
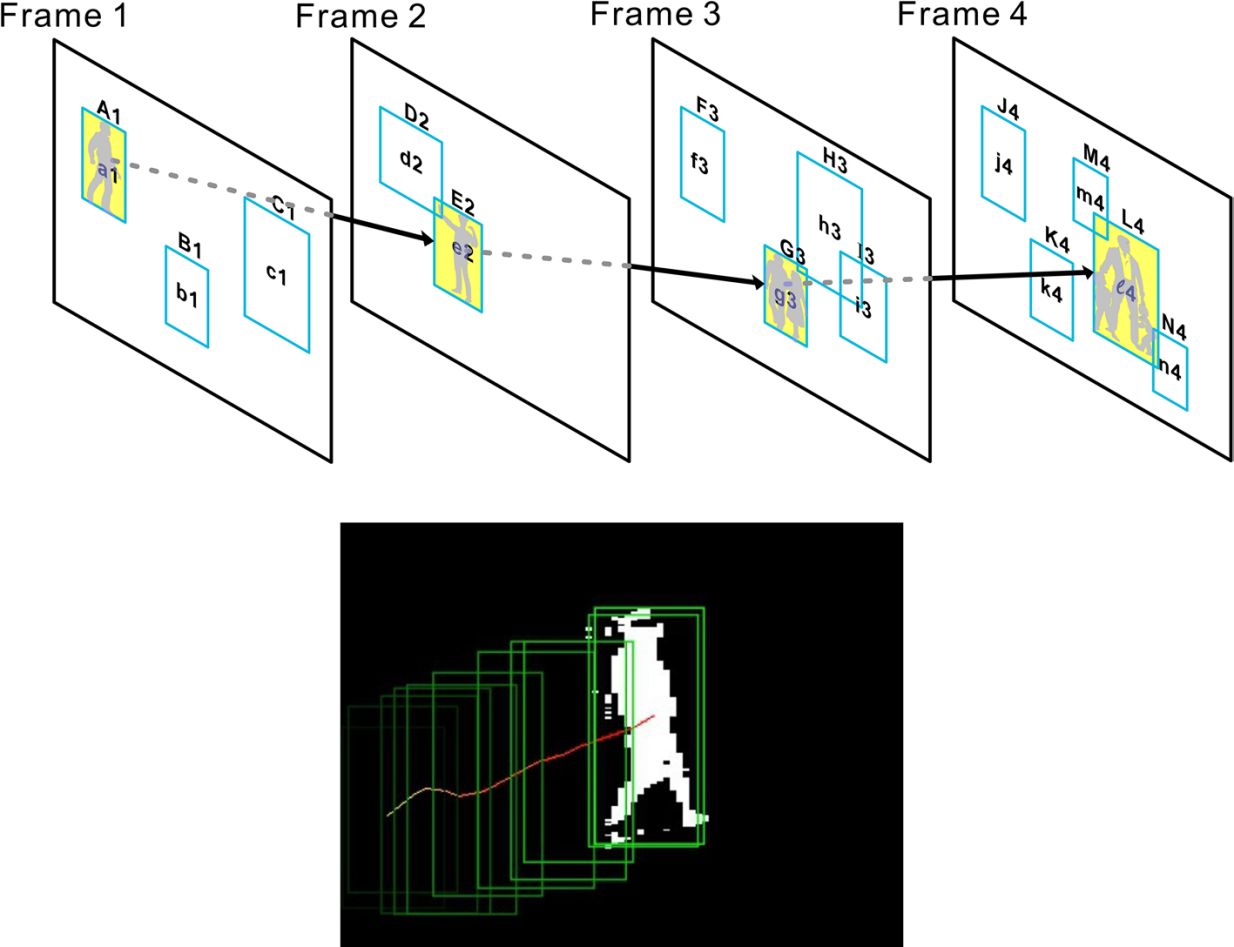
The Viterbi algorithm for finding the most probable human motion sequence. a_1_, e_2_, g_3_, and l_4_ represent the computed probabilities that bounding boxes A_1_, E_2_, G_3_, and L_4_ have a human presence, respectively. The green boxes in the bottom figure (actual, real human tracking example) represent the bounding boxes in sequence, collected from different frames.

We used the Viterbi algorithm to find a most probable human-presence path from the above. For instance, consider the path A_1_, E_2_, G_3_, L_4_ …. This particular path had a human presence probability of a_1_ × e_2_ × g_3_ × l_4_ × …. (whose log likelihood is log(a_1_) + log(e_2_) + log(g_3_) + log(l_4_) + …) if we assumed frame-level independence (which although not completely correct did work quite well in practice). Please see Fig. 9. Finally, we picked a group of candidate paths whose probabilities were the highest. Note that probabilities were needed here. As pointed out earlier, the inherent capability of RVM to estimate probabilities came as very convenient for our Viterbi algorithm step here. Note also that we enforced each picked path to also conform to spatial smoothness. In other words, each pair of adjacent subregions in a path (say D_2_ and G_3_) must not be spatially too far away from each other in the pixel space.

Finally, for each path in the group of candidate paths, we constructed a candidate video clip from the temporal subregions in the path. Note that the use of temporal subregions matched our temporal subregions of fall detector training process described earlier, which effected a consistent machine learning flow. We fed this subregion video clip to our fall detector. Whenever our fall detector detected a fall in any of these paths, our system triggered a fall alarm, captured the image from the offending sequence of frames from the path, and sent the image out for families or paramedics to take further action.

**Figure 10:**
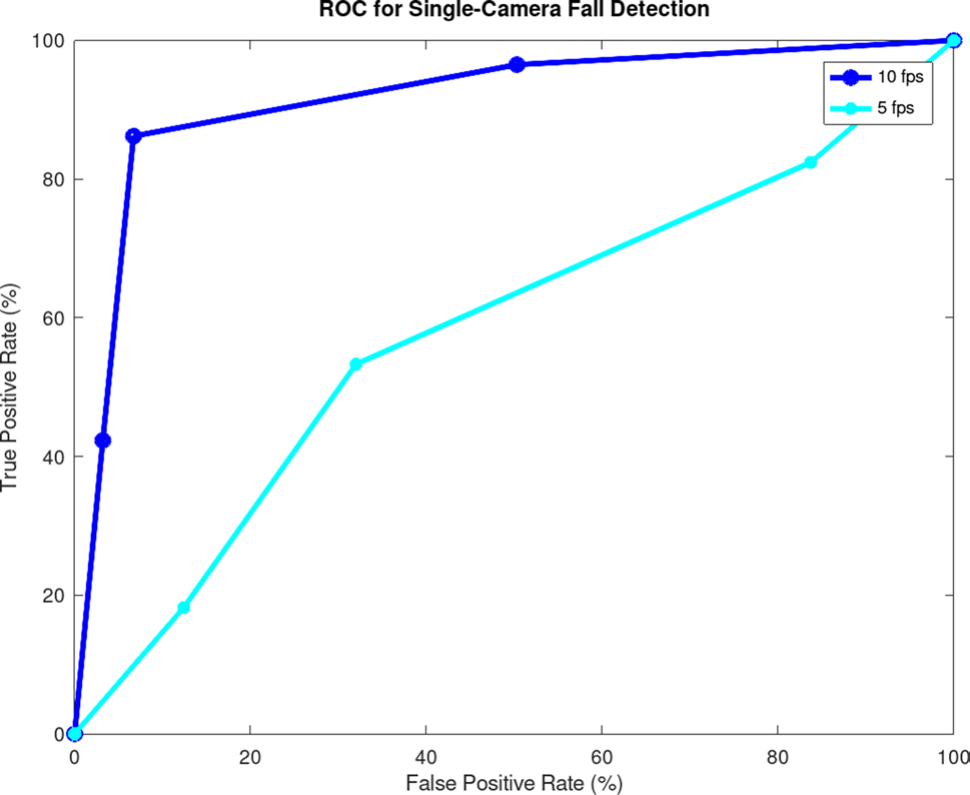
ROC performance metrics for different AI speeds in FPS for single-camera fall detection. Simply making AI faster, while all else being equal, has significant benefits on the overall performance.

Our human detector achieves a held-out accuracy of 89% and a training accuracy of 94%. Figure 10 shows the Receiver Operating Characteristic (ROC) curve of single-video fall detection accuracy, where the true positive rate (TPR) and false positive rate (FPR) are controlled via a tunable threshold τ, 0 < τ < 1, in which the emission probability *y* of fall as output by RVM is classified as fall if *y* > τ. In both cases (human detection and fall detection), the results are averaged over 10 random splits of the data into 80% training and 20% test set each, with equal weights applied to the positive and negative data instances. It is crucial to point out that these empirical statistical results of our human detector or even our fall detector, whilst sufficiently robust to warrant mass manufacturing, are not the primary focus of our work. Rather, our research reveals that speedy computation is key. Any instantiation of a lean, fast AI system, of which our proposed specifics are but one example, can potentially achieve equally good fall detection. Indeed, we find that when our system becomes sufficiently fast, obtaining high-accuracy results becomes much easier, if not altogether trivial. For instance, for a two- or three-camera setting, it is almost impossible for our system to miss a fall, which happens only if all cameras miss it. But it is precisely our speedy computation that grants us this luxury of multi-camera AI in an extremely affordable manner. This notion that computational efficiency, rather than statistical accuracy itself, lies at the heart of intelligence is perhaps best affirmed by Robert Schapire^59^: “It is often the case that a learning problem cannot be solved, even when more than enough data has been provided to ensure statistical generalization, solely because the associated computational problem is intractable”.

### Manually crafted rules

The advantages of using surveillance camera systems to detect falls are numerous. First, falls are less likely to be occluded by furniture because cameras are installed at high altitudes. Second, cameras and their viewing angles are less likely to be tampered. Third, they are widely available, affordable, and durable. The disadvantage is the complexity of the different fall scenarios implicated when cameras are installed at high altitudes, requiring an intricate AI analysis.

For the faller at a far distance from the camera mounted at a high altitude, different phenomena are observed in falls of different directions. For a sideway fall, the initial height (*H*_*0*_) viewed by the camera is reduced to *H*_*0*_* Cos*(*τ*), where τ is the upward angle from the person’s perspective, with the same base level after the fall. For a forward fall, the apparent height is also reduced to *H*_*0*_* Cos*(*τ*) but with a lowered base level after the fall, since the head becomes lower than the foot from the camera’s point of view. For a backward fall, the apparent height is also reduced to *H*_*0*_* Cos*(*τ*) with the same base level after the fall, but the final apparent height is higher than that of the sideway fall (as if the person fell on an inclined plane). As the camera distance becomes closer, the analysis becomes more complicated. To ensure optimal AI performance accuracy and efficiency, the above information is used to craft into rules to help the system better determine the fall.

## Supplementary Information

Supplementary Information

## Data Availability

All data generated or analyzed during this study are included in this article (and its Supplementary Information file).
