# Supplementary material for: An eight-camera fall detection system using human fall pattern recognition via machine learning by a low-cost android box
Source: Sci Rep. 2021 Jan 28;11:2471. doi: 10.1038/s41598-021-81115-9 (PMC7844246; doi:10.1038/s41598-021-81115-9)
Supplement: Supplementary file 1 — Supplementary Information [file 41598_2021_81115_MOESM1_ESM.pdf]

Supplementary Information

Title:

An Eight-Camera Fall Detection System Using Human Fall Pattern Recognition via Machine Learning by a Low-Cost Android Box

Authors:

Francy Shu, MD[1,*], Jeff Shu, MS[2]

[1]Assistant Professor
Division of Neuromuscular Medicine
Department of Neurology
University of California, Los Angeles Medical Center
300 Medical Plaza, B200
Los Angeles, CA, USA 90095
626-616-8538
fshu@mednet.ucla.edu

[2]Chief Technology Officer
SpeedyAI, Inc.
19940 Ridge Estate Ct
Walnut, CA, 91789
626-674-1777
jeff@speedyai.com

Table of Contents

Histogram of Oriented Gradients (HOG) Feature Extraction 3

Relevance Vector Machine (RVM) Learning Framework 6

Testing Data 7

Histogram of Oriented Gradients (HOG) Feature Extraction

Consider first a tiling of an image (a video frame) into a rectangular grid of cells, as shown in Supplementary Figure S1. Each cell is associated with it 25 bins whose values are initialized to 0. The purpose of these 25 bins will be addressed shortly. Spatially, each cell also consists of some number of pixels, and the insert in Figure S1 shows one such example in magnified view. For a pixel, we approximate its horizontal and vertical intensity value changes as (Δ_x, Δ_y) = (R - L, T - B), where L, R, T, and B are the intensities of the left, right, top, and bottom spatial neighbors of the pixel, respectively (Supplementary Fig. S2). With this (Δ_x, Δ_y), we compute a dot product with each of the 8 unit vectors shown in Supplementary Figure S3a representing 8 unsigned directions, and a dot product with each of the 16 unit vectors shown in Supplementary Figure S3b representing 16 signed directions, 8 of which are essentially the opposite of the 8 unsigned directions. Lastly, we note the direction corresponding to the unsigned case whose dot product has maximal magnitude, and the direction corresponding to the signed case whose dot product has maximal value. For ease of ongoing discussion, for the pixel under consideration, say the unsigned direction chosen is 67.5° and the signed direction chosen is 247.5°. Let $m_{\text{orientation}}$ be this maximal magnitude, which is the absolute value of the dot product with the 67.5° or the 247.5° unit vector. Finally, let m_{texture} be the sum of the absolute values of all the 8 dot products from the unsigned directions. The choice of names "orientation" and "texture" will be discussed shortly.

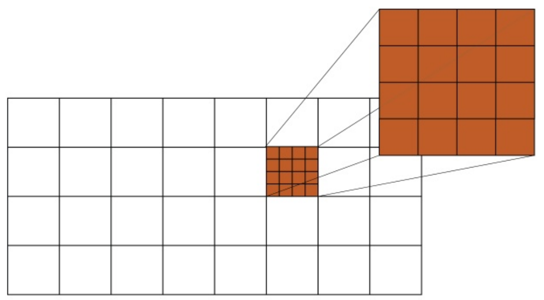

Fig. S1 Image tiling into cells. Insert: Each cell consists of some number of pixels.

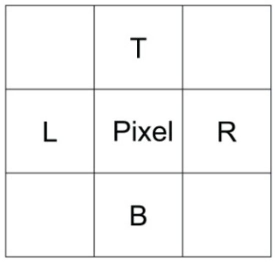

Fig. S2 The intensities of the left (L), right (R), top (T), and bottom (B) spatial neighbors used to calculate pixel intensity differential.

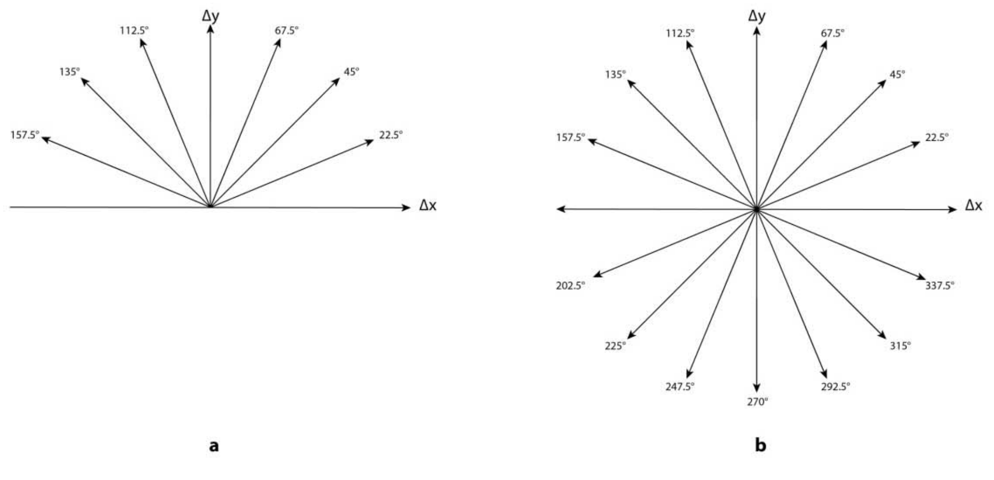

Fig. S3 HOG orientation directions. a, Eight unit vectors representing eight unsigned directions. **b,** Sixteen unit vectors representing sixteen signed directions, eight of which are essentially the opposite of the eight unsigned directions.

Imagine for now that each of the 25 bins of a cell has a name. Eight of the names are unsigned 0°, unsigned 22.5°, …, unsigned 157.5°. Sixteen of the names are signed 0°, signed 22.5°, …, signed 337.5°. One of the names is texture. Then, for the cell *C* in which the pixel in the example above resides, we increment its bin values in the following manner.

bin[unsigned 67.5°] ←bin[unsigned 67.5°] + $m_{\text{orientation}}$

bin[signed 247.5°] ←bin[signed 247.5°] + $m_{\text{orientation}}$

bin[texture] ←bin[texture] + m_{texture}

Note that the unsigned and signed directions chosen in the manner above must always be identical or differ by 180°. The proof is straightforward and is hereby omitted. We traverse through the pixels in the image and compute the bin values as depicted above. Each cell's bins will be incremented for multiple times as a cell typically consists of multiple pixels. Therefore, these bin values are not necessarily sparse.

An image typically consists of three channels representing its color information. In the HOG feature extraction algorithm above, it is not specified which channel the pixel intensity value comes from. There are two possibilities here. First, we carry out the algorithm three times, once for each color channel, and for each pixel simply collect our $m_{\text{orientation}}$ and m_{texture} from whichever channel that would give the maximum value. Let us denote this possibility by Grayscale HOG, where each cell has 25 bins forming effectively a 25-vector. Second, we keep all the three outcomes and leave them intact, effectively expanding the bin size of each cell to 25 × 3 = 75. Let us denote this possibility by Color HOG, where the bins of each cell are essentially a 75-vector.

It is worthwhile to pause here and consider the intuitive motivation of the above algorithm as reflected from the naming convention. We note that $m_{\text{orientation}}$ is collected from the maximal dot product magnitude, which corresponds to the orientation in which the signal response is the strongest, thereby capturing the intuitive notion of orientation, a crucial ingredient for shape and general fall pattern. Furthermore, m_{texture} captures the magnitude of all orientations. Without favoring any orientation, it captures an intuitive, if rough, notion of texture where patch surface appearance and consistency matter. In Grayscale HOG, we reduce information from three color channels to effectively only one, mimicking a "grayscale" image. Of course, it is very different from the conventional notion of reducing an RGB image to a grayscale one. The name here is for analogous purpose only. Finally, in Color HOG, we genuinely retain the information of each color channel (in the form of HOG features), which is useful for us to assess how color and lack thereof affect the performance of our system.

We now convert the h-vector, where h = 25 or 75, of each cell into a single discrete value known as a texton. Consider first a (large) set of HOG features of h-vectors collected as described above over each frame of every video in the training data set. We apply the standard k-means clustering algorithm to obtain k = 400 clusters for this set of h-vectors. Each cluster corresponds to a specific texton, and we can use these 400 clusters as a dictionary to assign any h-vector to the specific texton by determining its closest cluster in the Euclidean $\mathbb{R}^h$ space. Now, any rectangular subregion R of an image is simply a tiling of textons as shown in Supplementary Figure S4. Consider the entire region $R_1 = R$ as shown in Supplementary Figure S5a, the 4 equally partitioned regions R_2, R_3, …, R_5 in Supplementary Figure S5b, and the 16 equally partitioned regions R6, R7, …, R21 in Supplementary Figure S5c, all from the same image. In each of these 21 regions, we tabulate its residing textons into a 400-histogram representing the frequency of occurrence of each texton in the region. Concatenating these histograms, we see that any rectangular subregion can be represented as a 21 × 400 = 8400-vector. Finally, the histogram intersection function is applied to pairwise 8400-vectors to form a type of spatial pyramid kernel[1], which can be fed directly to the RVM. This establishes our image semantics-based feature engineering.

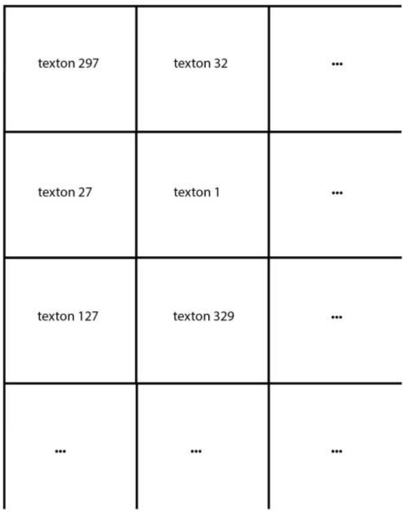

Fig. S4 Grid of textons. Their occurrence histograms are used as image semantics-based features.

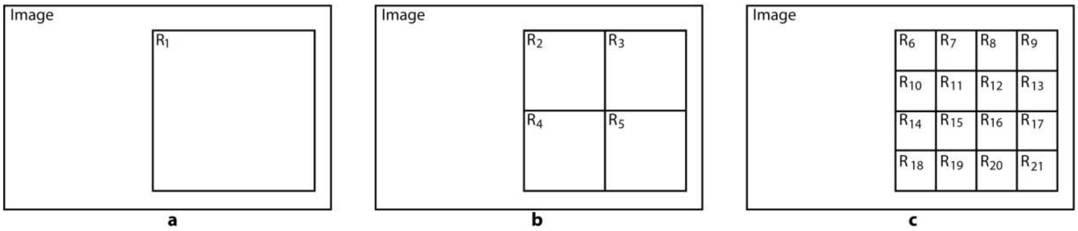

Fig. S5 Spatial pyramid of histograms. They are essentially spatially-aware bags of features.

It is important to note that numbers such as 8 (the number of discrete orientations) or 400 (dictionary size) are tunable parameters. We use concrete numbers in our discussion for only the purpose of ease of understanding and avoidance of algebraic notational clutter, without any loss of generality.

Relevance Vector Machine (RVM) Learning Framework

We now start RVM[2] to train our detectors, with details of the learning framework as follows. Consider first the extracted features represented as the design matrix $\mathbf{\Phi}$ of size $N \times M$, where N is the number of training instances and M is the number of features. Denote the jth column by ϕ_j, $1 \leq j \leq M$. We seek to find a preferably sparse M-vector $\mathbf{w}$ for which $y = \sigma\left(\mathbf{w}^{\mathrm{T}}\phi(\mathbf{x})\right)$, where $\phi(\mathbf{x})$ is a feature vector, would approximate the probability that the data $\mathbf{x}$ is ham (human/a falling event) rather than spam (not human/not a falling event), with sigmoid function $\sigma(u) \doteq \frac{1}{1+\exp(-u)}$ ensuring the value of y to lie in the range $(0, 1)$ so that $\mathbf{x}$ would be classified as ham if, for example, $y > 0.5$.

Consider a zero-mean Gaussian prior $p(\mathbf{w}|\boldsymbol{\alpha})$, where for each w_j of $\mathbf{w}$ the M-vector hyperparameter $\boldsymbol{\alpha}$ satisfies $w_j \sim \mathcal{N}\left(0, \alpha_j^{-1}\right)$ for each *j*. Let $t \in \{0, 1\}$ be the ham/spam ground truth indicator variable (for each training instance). As there is no known polynomial-time algorithm for maximizing the posterior $l \doteq p(\mathbf{w}|\mathbf{t}, \boldsymbol{\alpha})$ over $\mathbf{w}$, we follow a standard aproximation technique of using iterative reweighted least squares (IRLS) to find a local optimum using iterative updates akin to the second-order Newton method with gradient $\nabla \ln l = \mathbf{\Phi}^{\mathrm{T}}(\mathbf{t} - \mathbf{y}) - \mathbf{A}\mathbf{w}$ and Hessian $\nabla\nabla \ln l = -\left(\mathbf{\Phi}^{\mathrm{T}}\mathbf{B}\mathbf{\Phi} + \mathbf{A}\right)$, where $\mathbf{A}$ is the diagonal matrix of $\boldsymbol{\alpha}$ and $\mathbf{B}$ is an $N \times N$ diagonal matrix with entries $y_i(1 - y_i), 1 \leq i \leq N$, yielding upon proper convergence $\mathbf{w}^{\mathrm{IRLS}} = \mathbf{A}^{-1}\mathbf{\Phi}^{\mathrm{T}}(\mathbf{t} - \mathbf{y})$ and $\Sigma^{\mathrm{IRLS}} = \left(\Phi^{T}\mathrm{B}\Phi + \mathrm{A}\right)^{-1}$.

Maximizing the evidence function $p(\mathbf{t}|\boldsymbol{\alpha}) = \int_{\mathbf{W}} p(\mathbf{t}|\mathbf{w})p(\mathbf{w}|\boldsymbol{\alpha})d\mathbf{w}$ by setting its derivative with respect to α_j to 0, we obtain the update rule $\alpha_j^{\text{new}} \leftarrow \frac{\gamma_j}{\left(w_j^{\text{IRLS}}\right)^2}$, where $\gamma_j \doteq 1 - \alpha_j^{\text{old}} \Sigma_{j,j}^{\text{IRLS}}$. Now, by defining $\hat{\mathbf{t}} \doteq \boldsymbol{\Phi}\mathbf{w}^{\text{IRLS}} + \mathbf{B}^{-1}(\mathbf{t} - \mathbf{y})$ and $\mathbf{C} \doteq \mathbf{B} + \boldsymbol{\Phi}\mathbf{A}\boldsymbol{\Phi}^{\text{T}}$, the classification problem is reduced to a regression one in which the usual quality $\mathbf{Q}$ and sparsity $\mathbf{S}$ parameters are readily evaluated as $q_j = \boldsymbol{\phi}_j^{\text{T}}\mathbf{C}^{-1}\hat{\mathbf{t}}$ and $s_j = \boldsymbol{\phi}_j^{\text{T}}\mathbf{C}^{-1}\boldsymbol{\phi}_j$, where $1 \leq j \leq M$. Finally, we drop any feature j for which $q_j^2 \leq s_j$, as it implies a divergent hyperparameter α_j that bounds w_j around 0 with arbitrary precision. This effectively prunes the jth feature away and induces sparsity on $\mathbf{w}$.

In summary, in each RVM iteration, we first run a number of subiterations for IRLS and one single update of the quality $\mathbf{Q}$ and sparsity $\mathbf{S}$ parameters as usual. Then, this RVM iteration is repeated for a number of times until convergence. Upon completion, $\mathbf{w}$ tends to be sparse, which results in efficient inference on the unseen data. Note that all features are considered in each RVM iteration, so a pruned feature in one iteration may be reacquired in a later iteration. In the above learning framework, we largely omit in the discussion the index i over the instances of training data to reduce notational clutter.

References

1 Lazebnik, S., Schmid, C., & Ponce, J. Beyond Bags of Features: Spatial Pyramid Matching for Recognizing Natural Scene Categories. `https://inc.ucsd.edu/~marni/Igert/Lazebnik_06.pdf` (accessed 1/1/2020).

2 Tipping, M. E. Sparse Bayesian learning and the relevance vector machine. *Journal of Machine Learning Research* **1**, 211–244 (2001).

Supplementary Testing Data Figure a-w

a, Stumbling test on Camera One at 2 meters (Part 1)

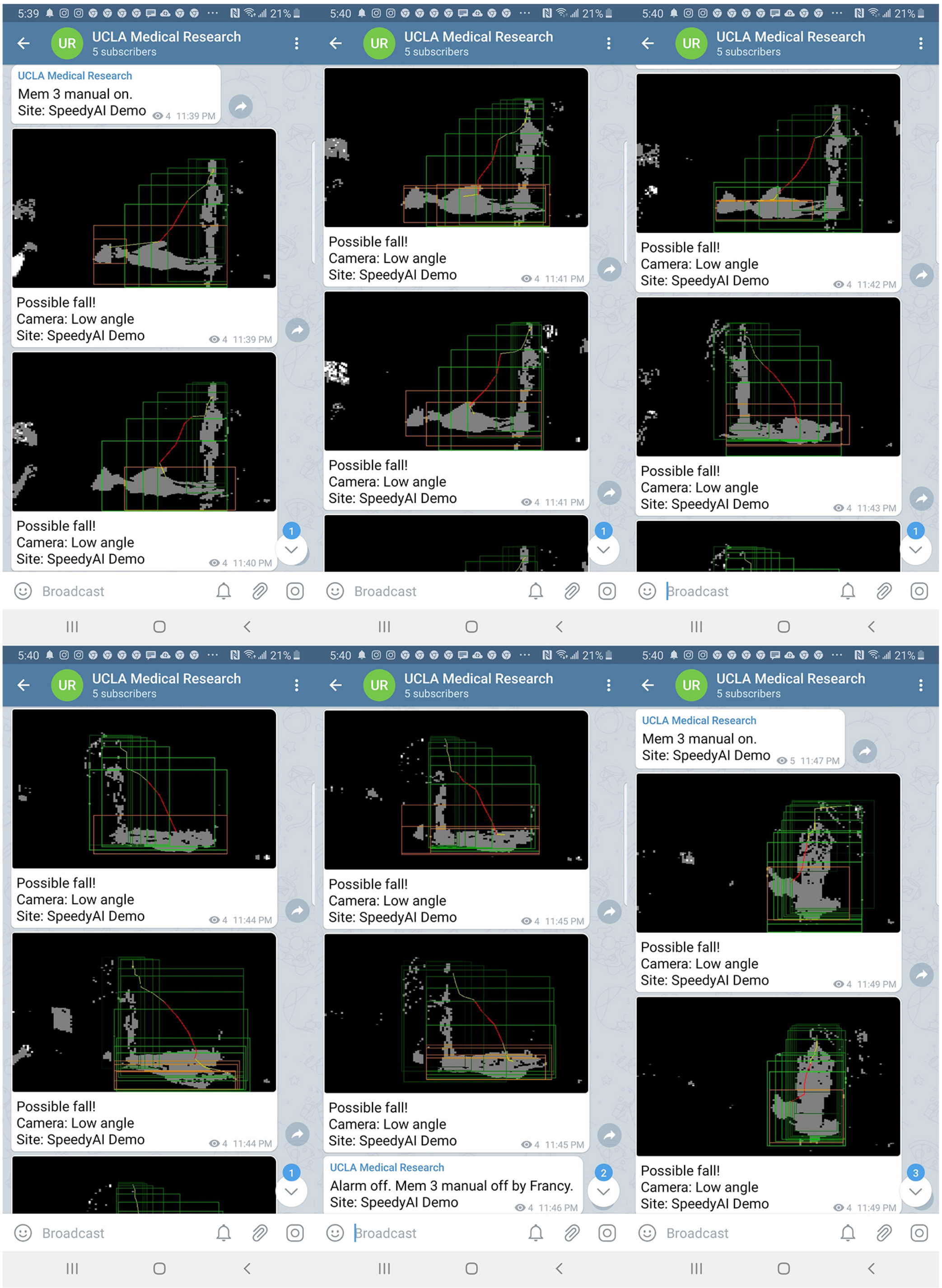

b, Stumbling test on Camera One at 2 meters (Part 2)

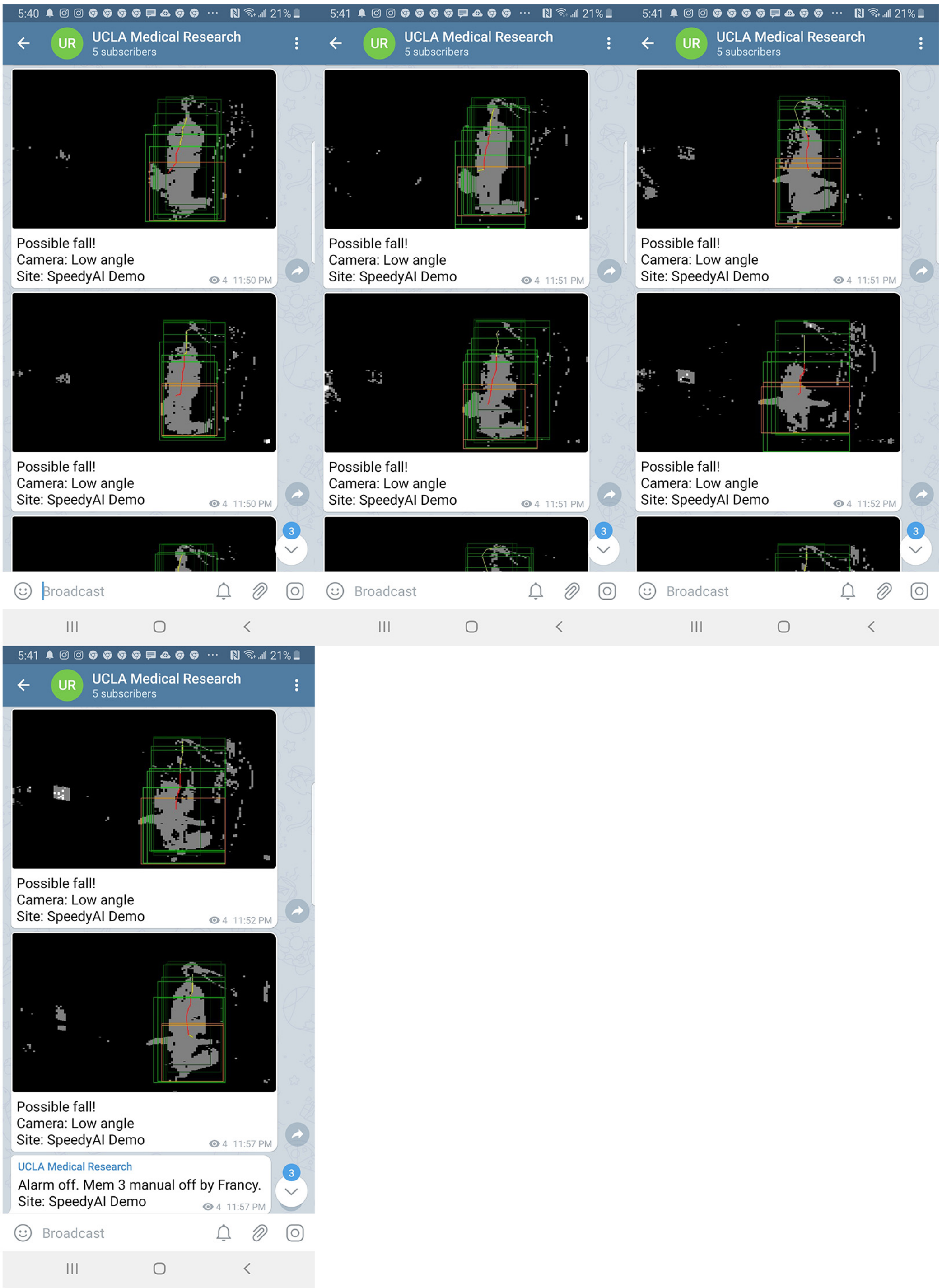

c, Stumbling test on Camera One at 5 meters (Part 1)

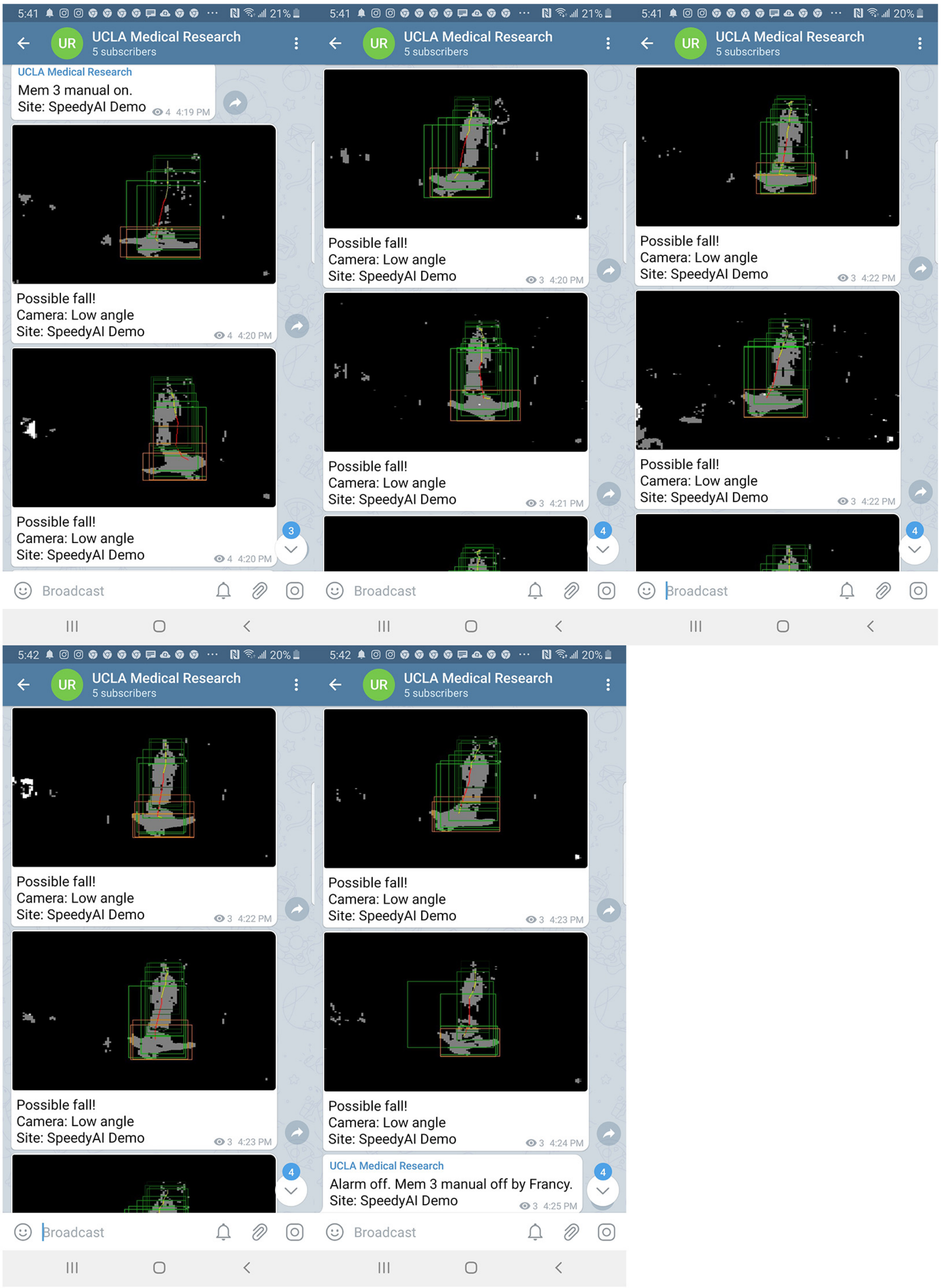

d, Stumbling test on Camera One at 5 meters (Part 2)

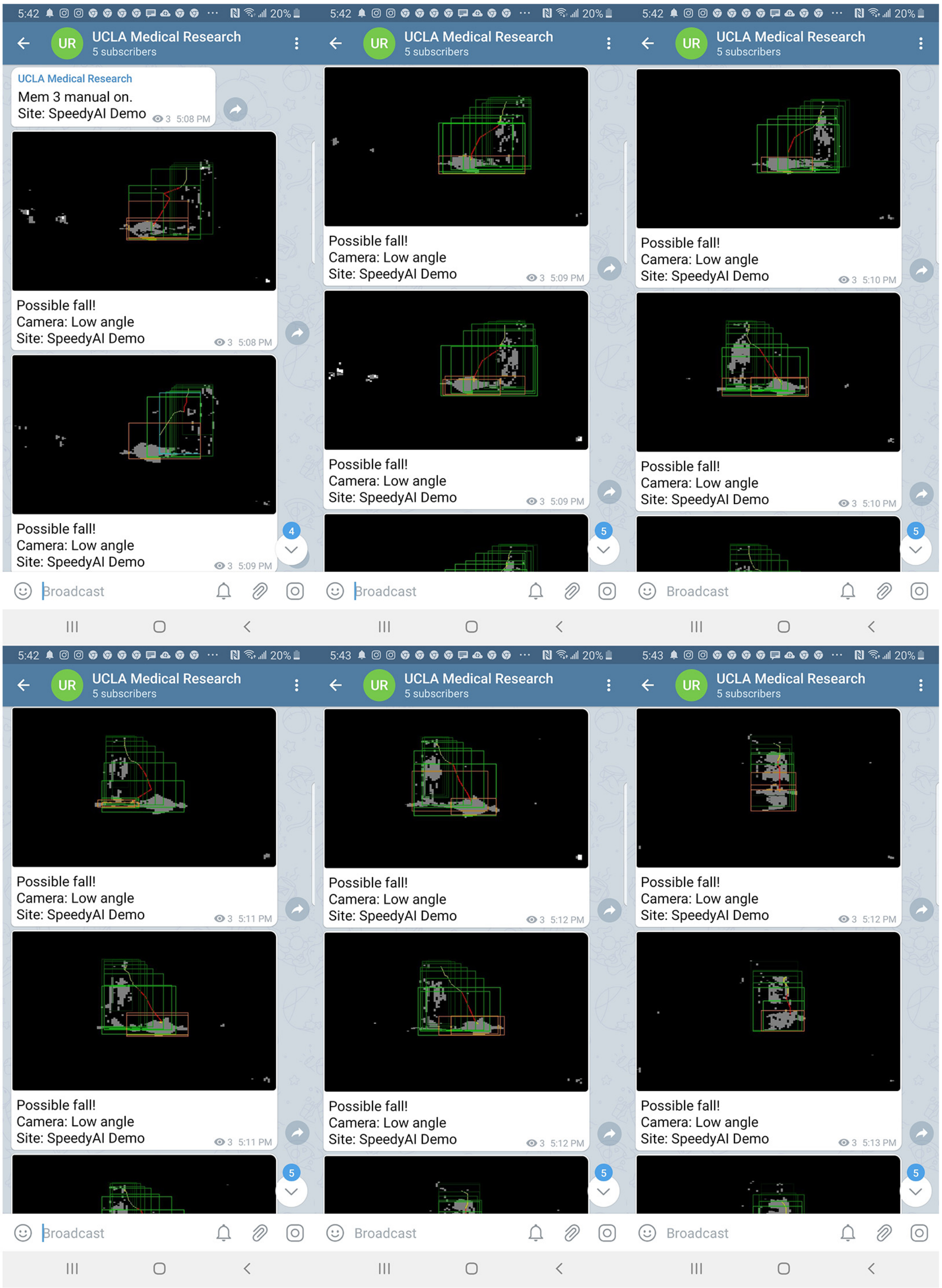

e, Stumbling test on Camera One at 5 meters (Part 3)

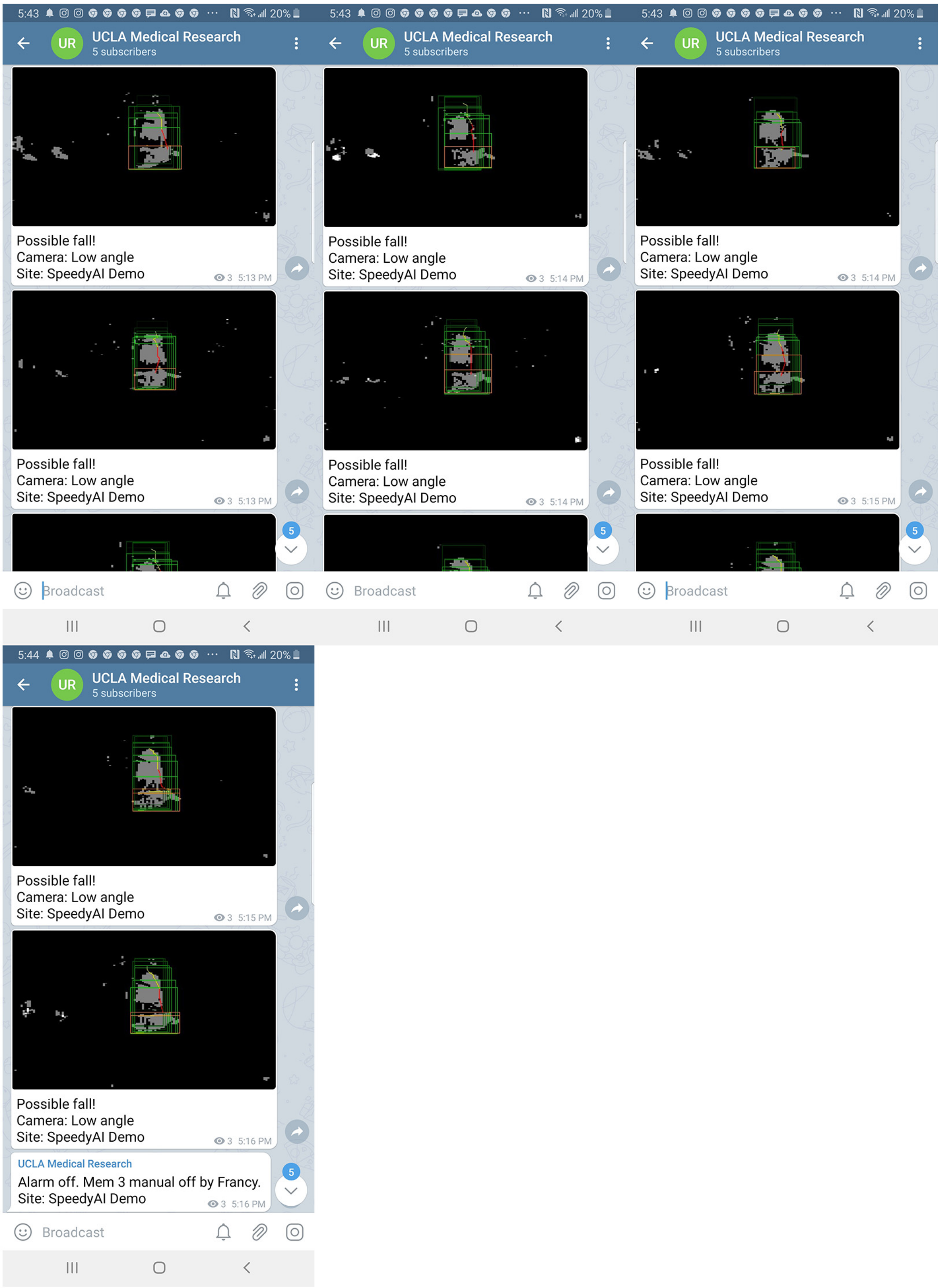

f, Stumbling test on Camera One at 8 meters (Part 1)

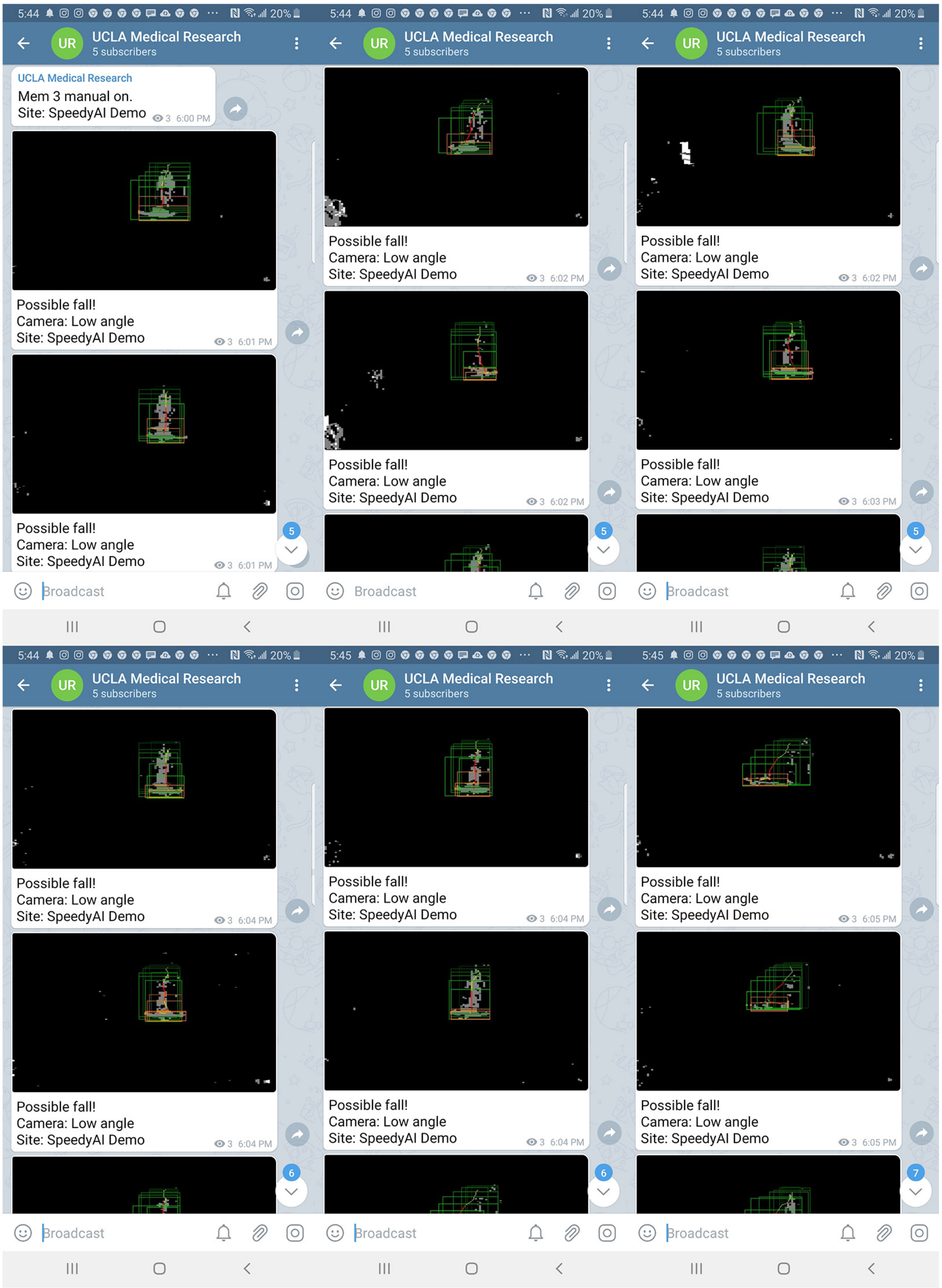

g, Stumbling test on Camera One at 8 meters (Part 2)

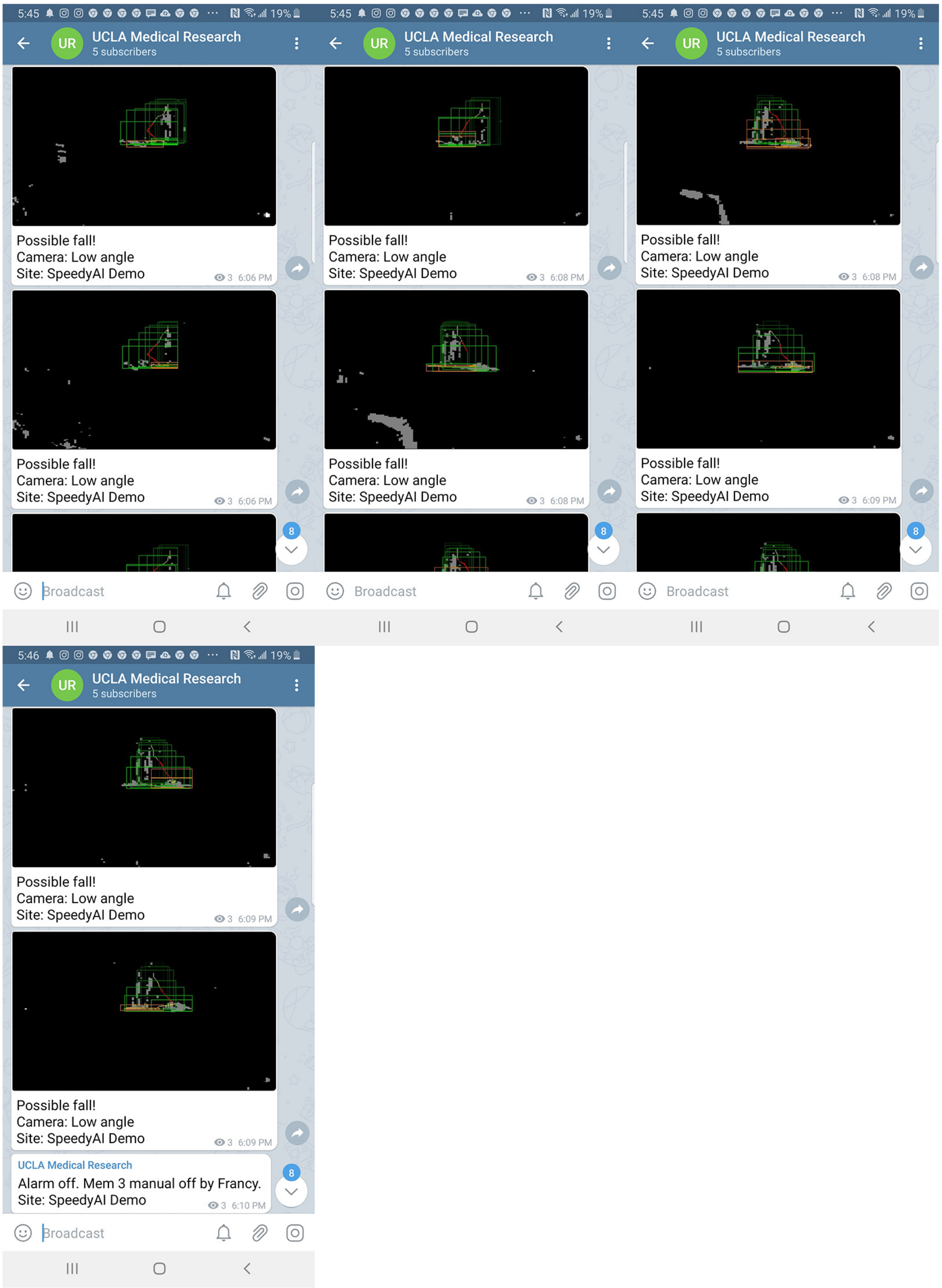

h, Stumbling test on Camera Two at 3 meters (Part 1)

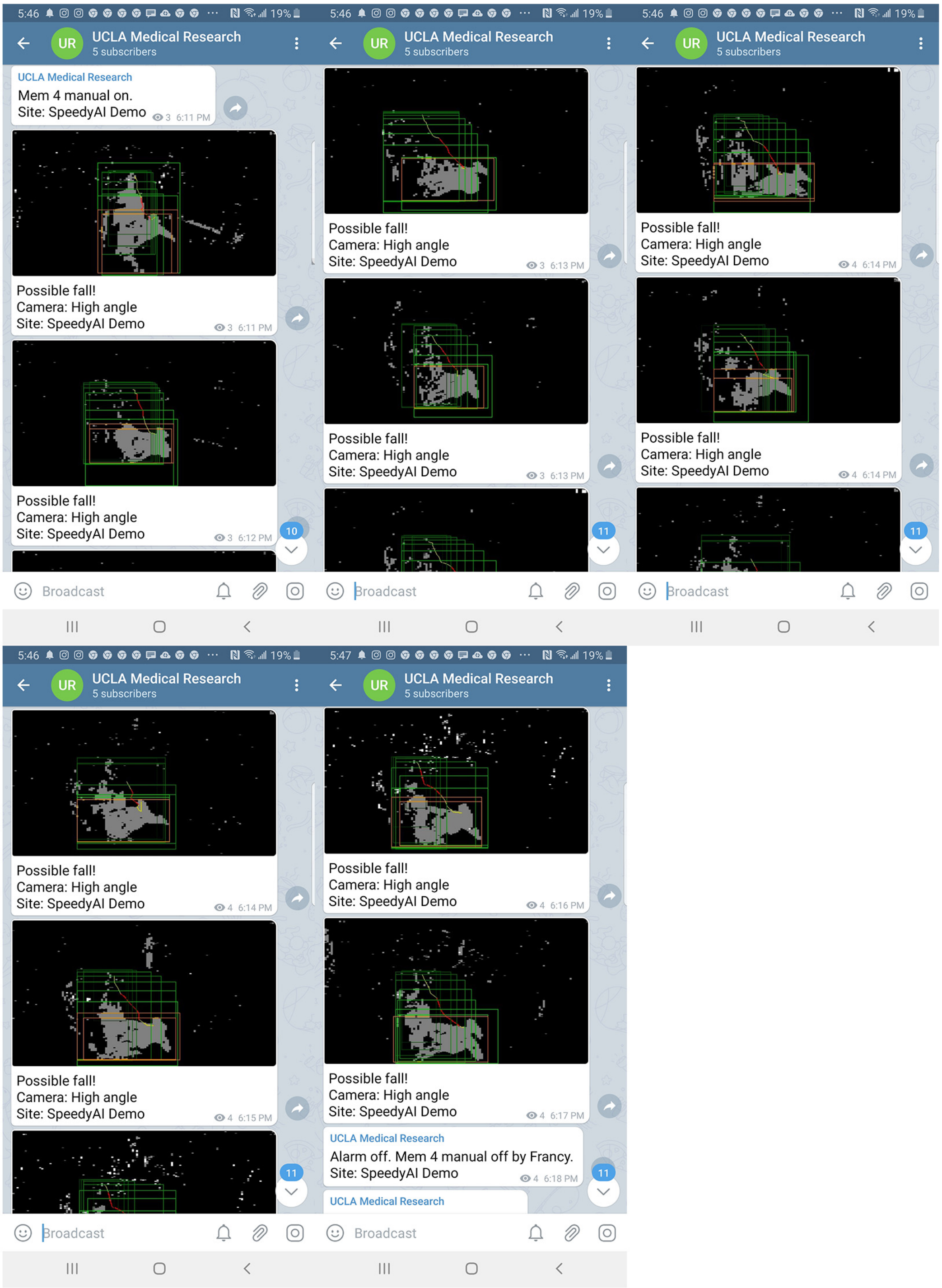

i, Stumbling test on Camera Two at 3 meters (Part 2)

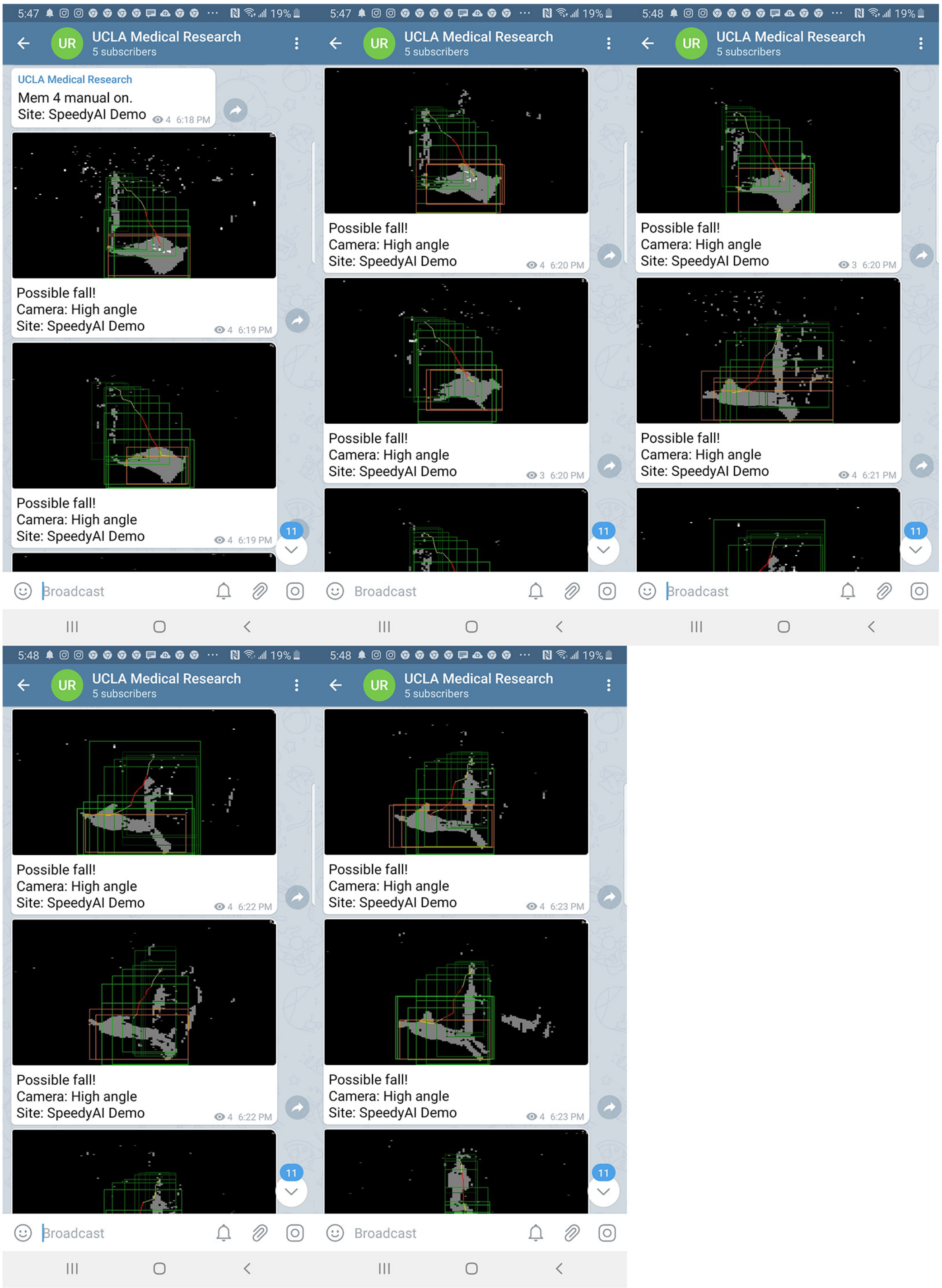

j, Stumbling test on Camera Two at 6.5 meters (Part 1)

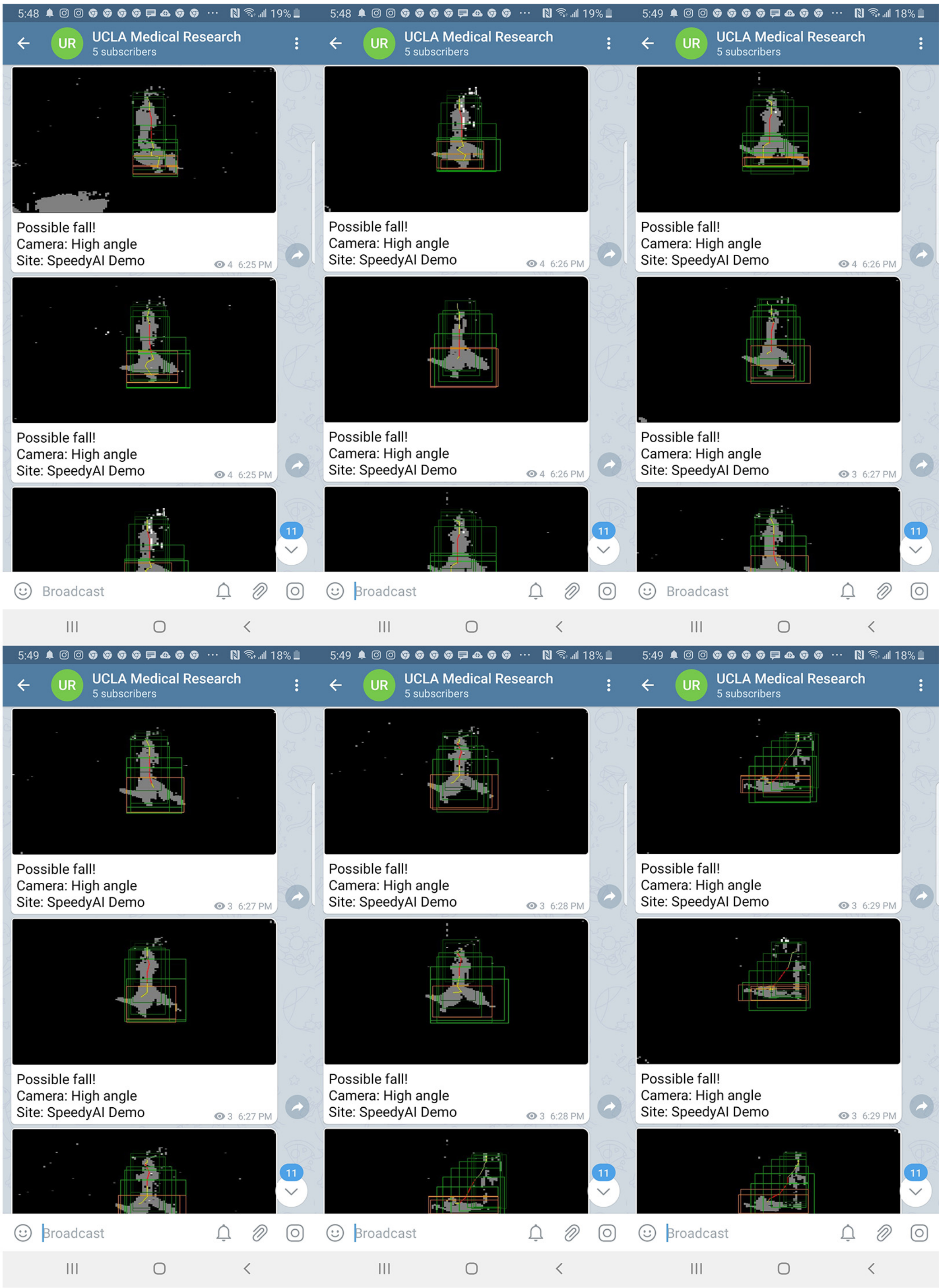

k, Stumbling test on Camera Two at 6.5 meters (Part 2)

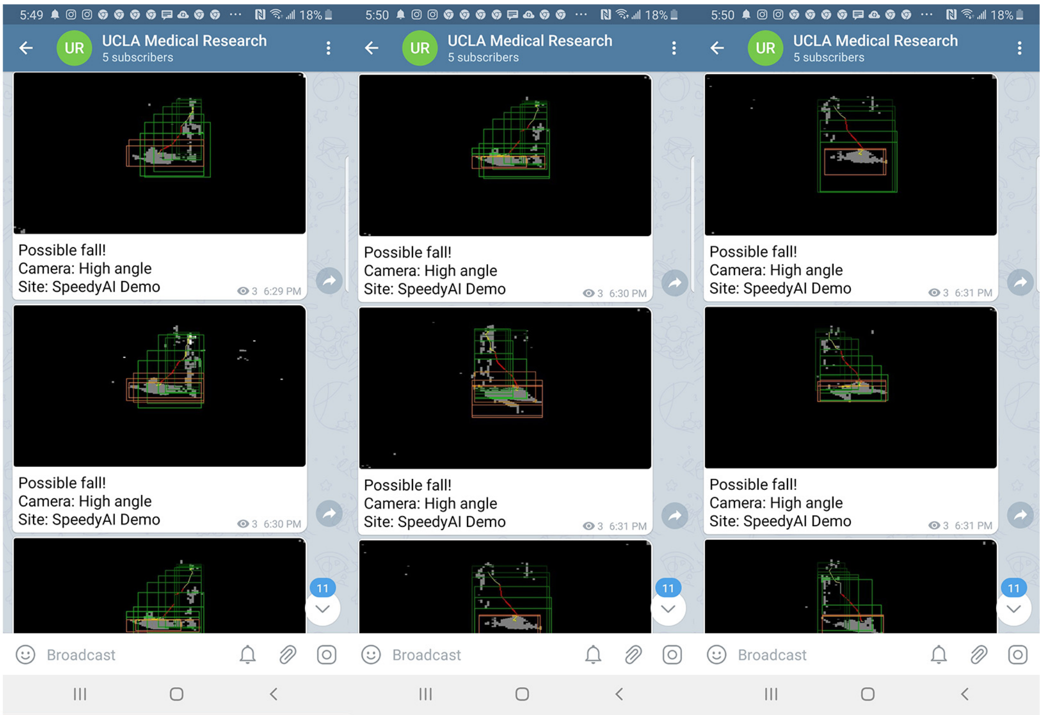

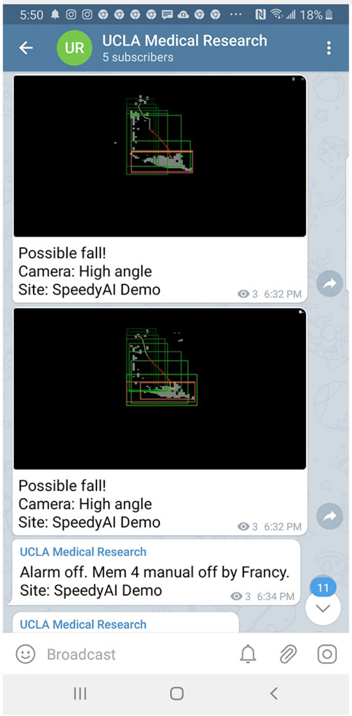

I, Stumbling test on Camera Two at 6.5 meters (Part 3)

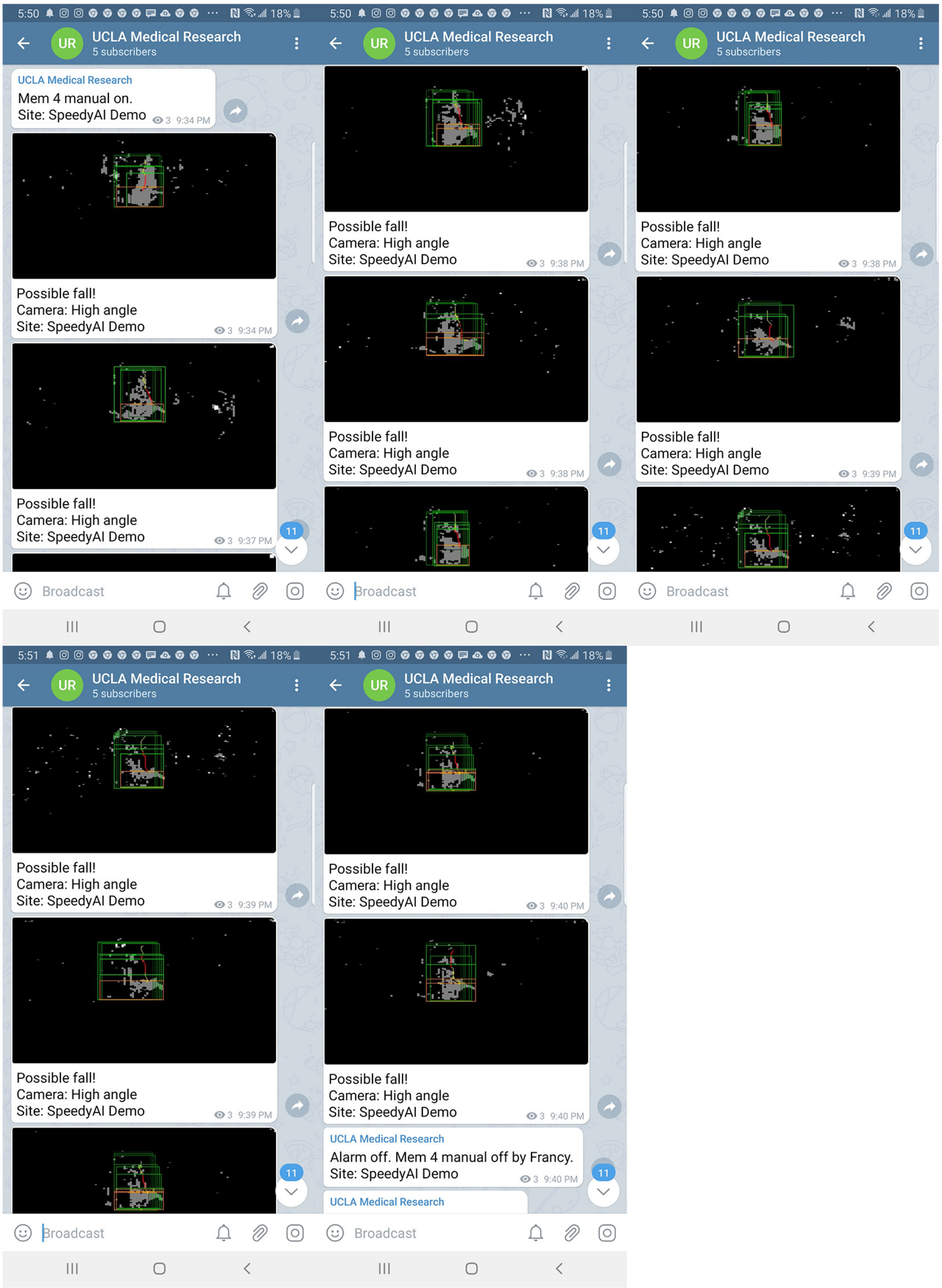

m, Stumbling test on Camera Two at 10 meters

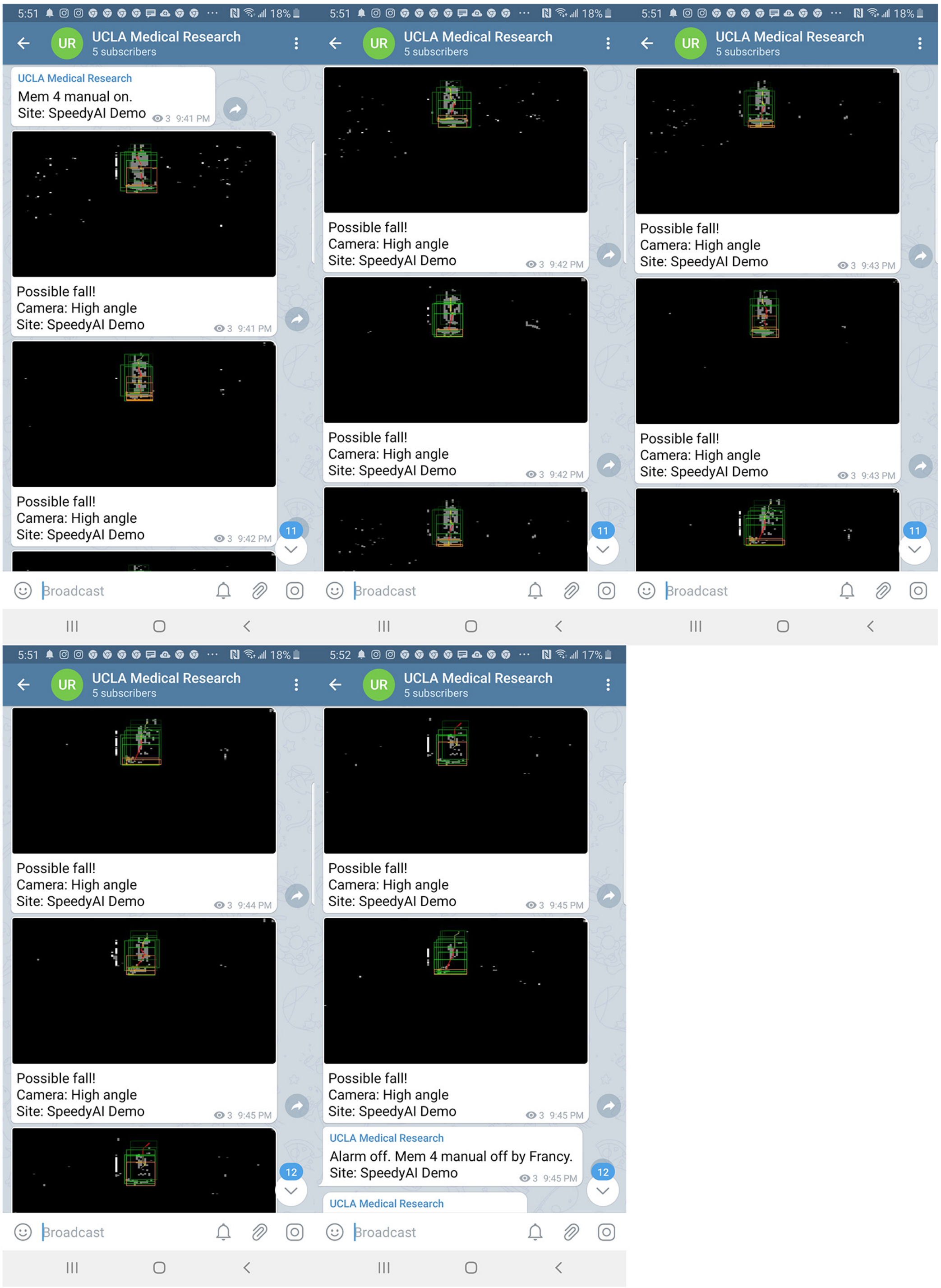

n, Slipping test on Camera One at 5 meters

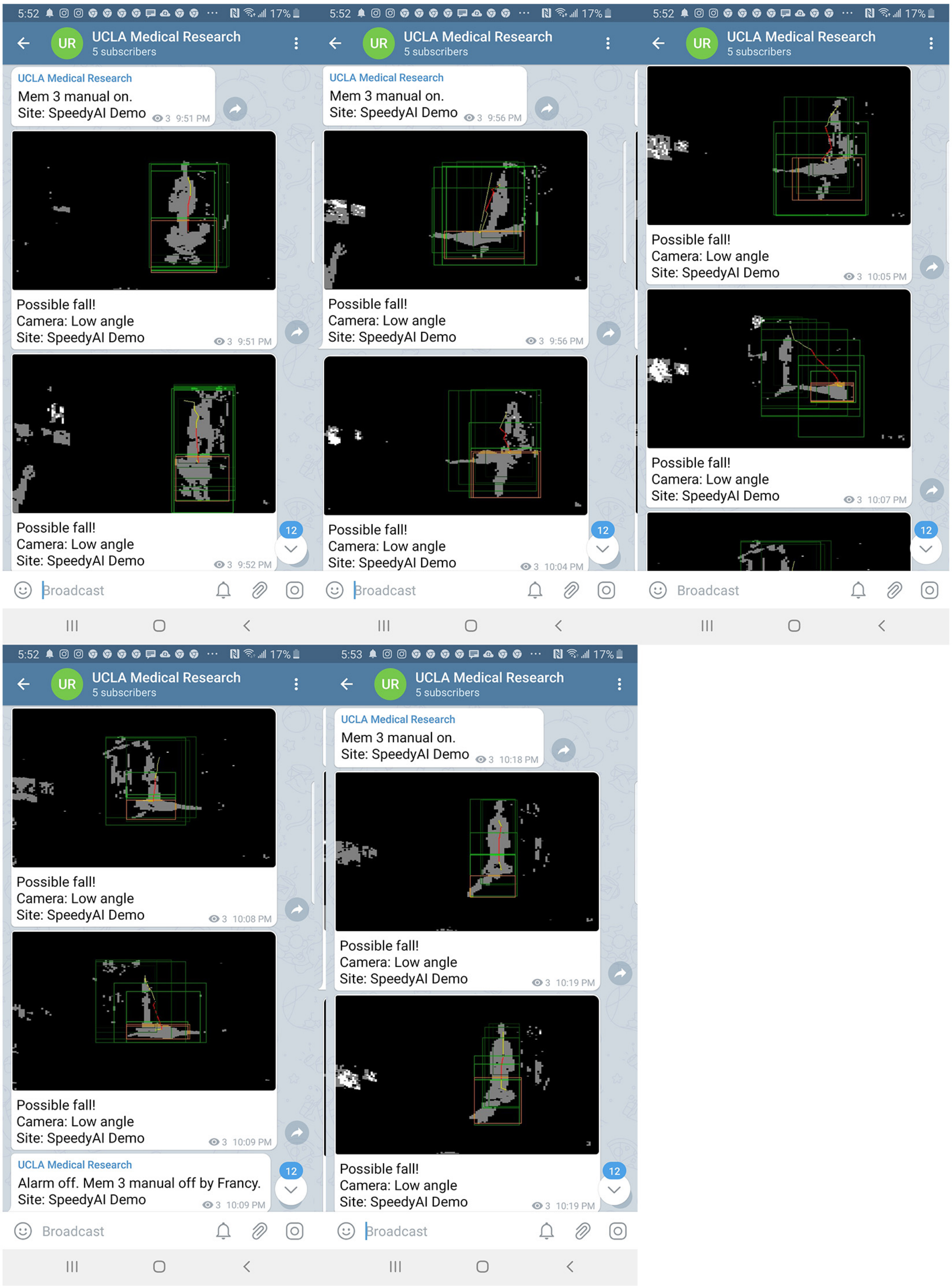

o, Fainting test on Camera One at 5 meters

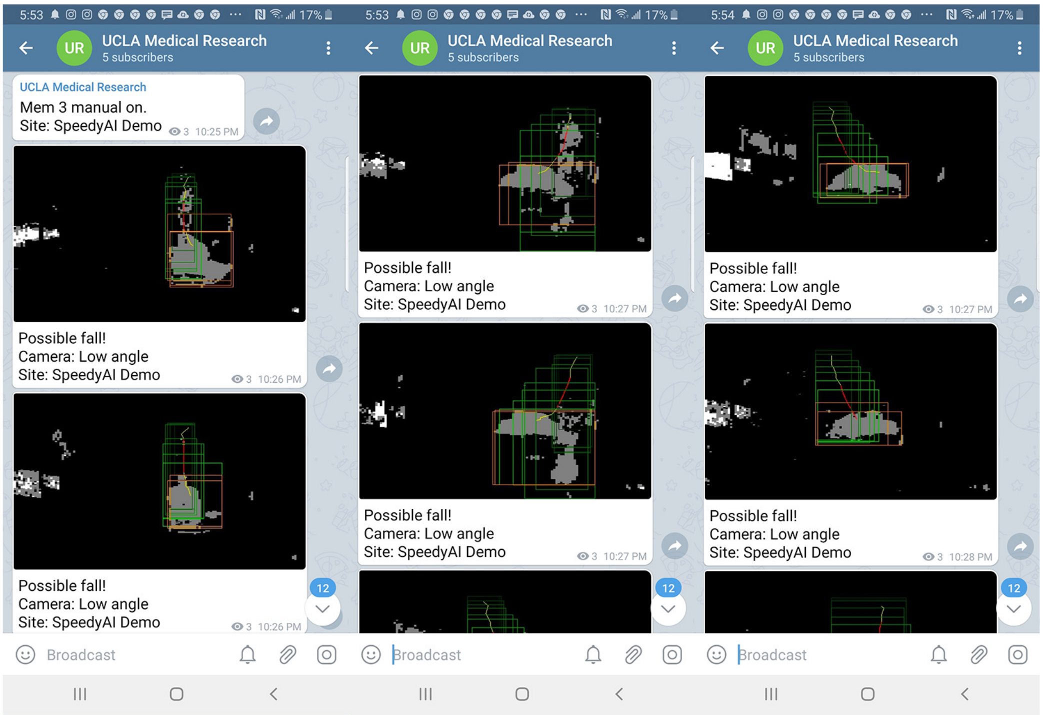

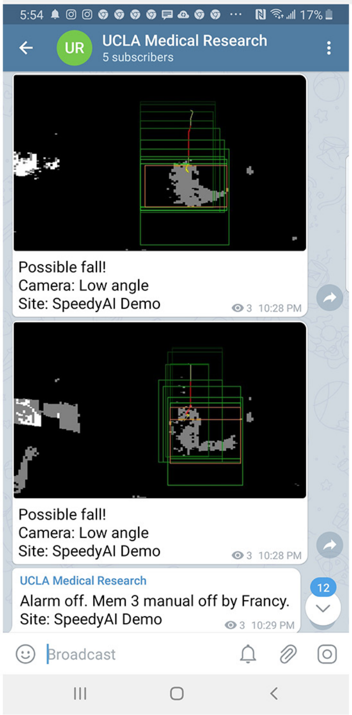

p, Falling off a ladder test on Camera One at 6 meters

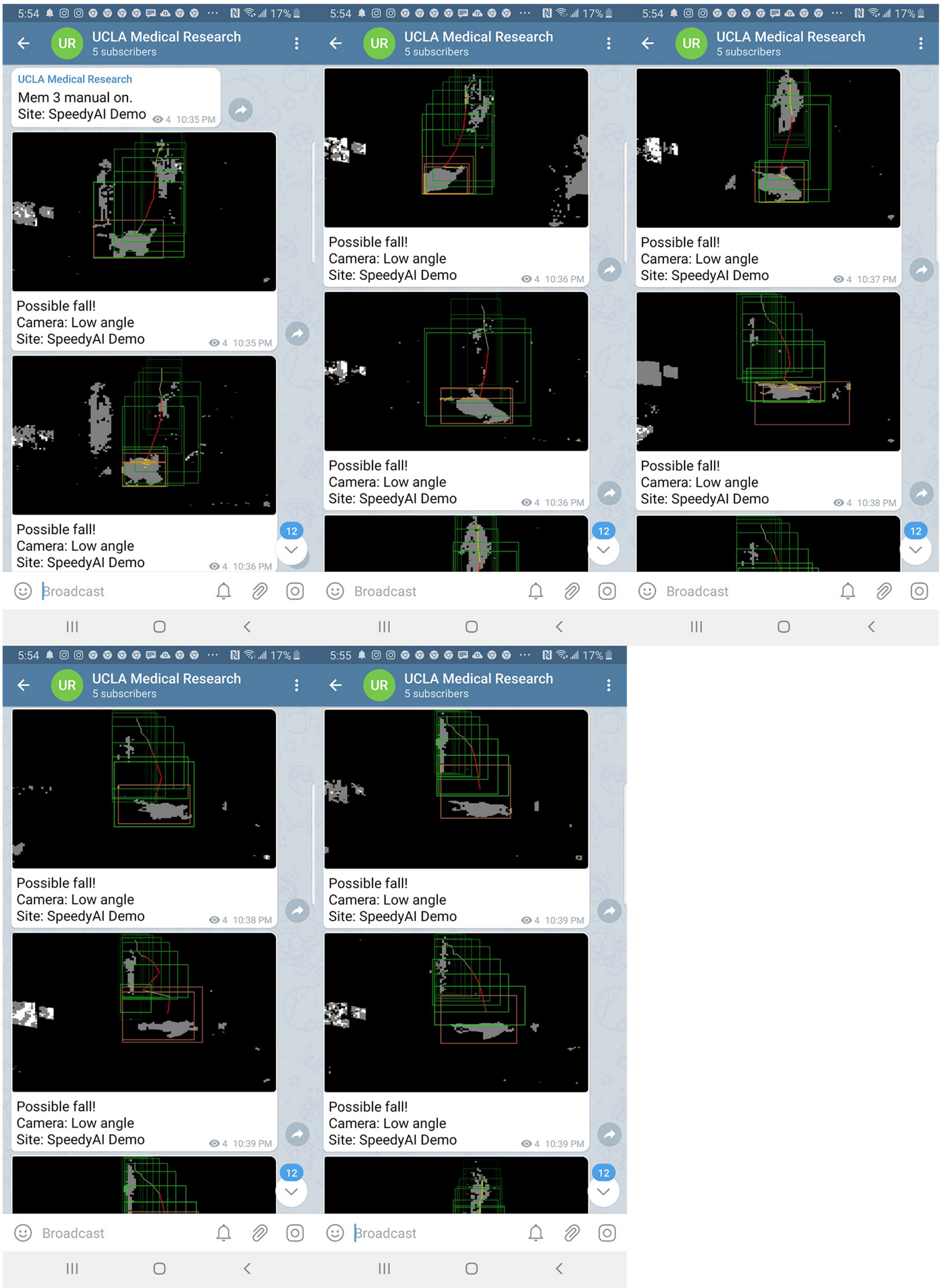

q, Jumping down from a desk and falling test on Camera One at 6 meters

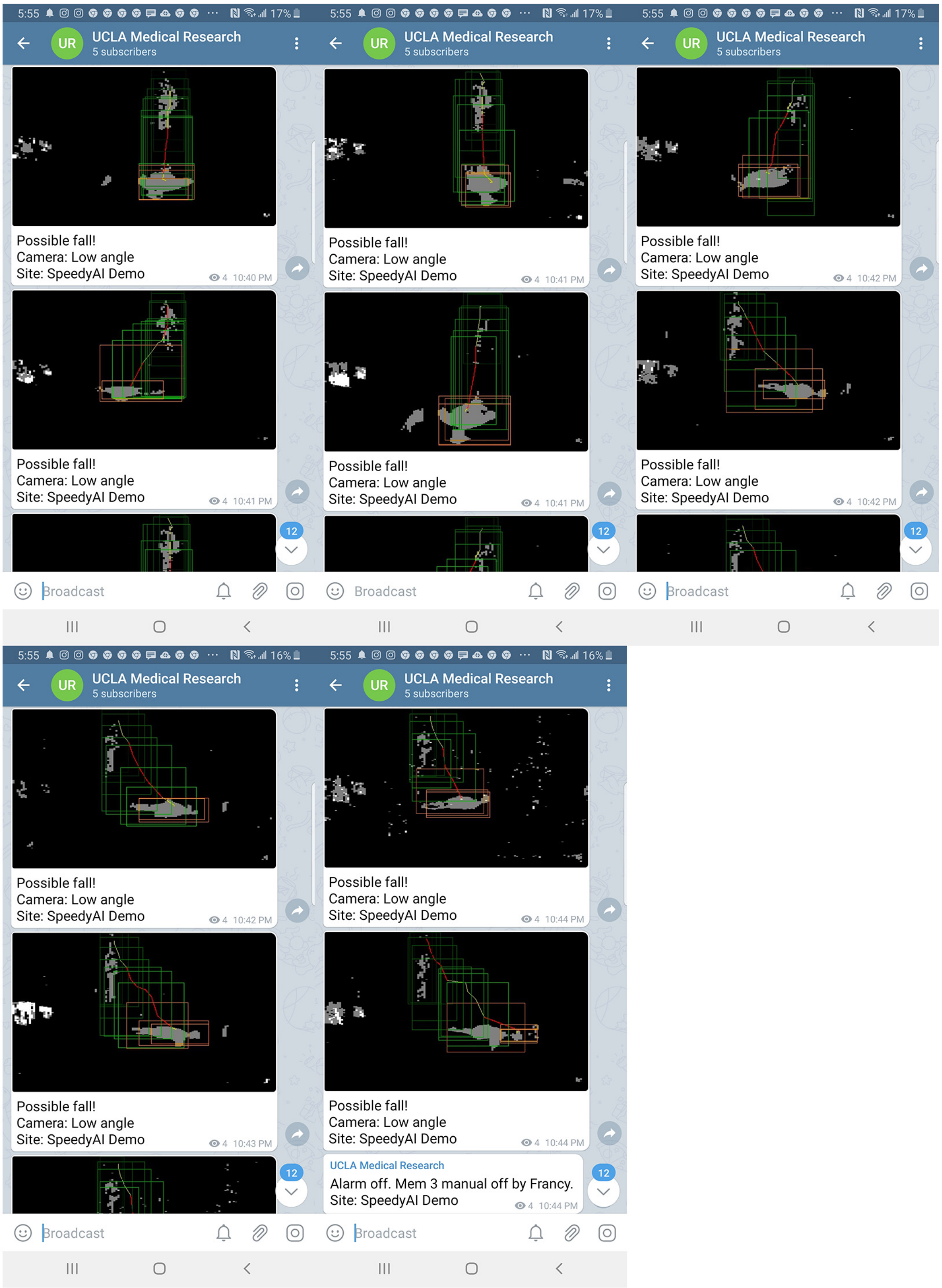

r, Collapsing upon standing test on Camera One at 5 meters

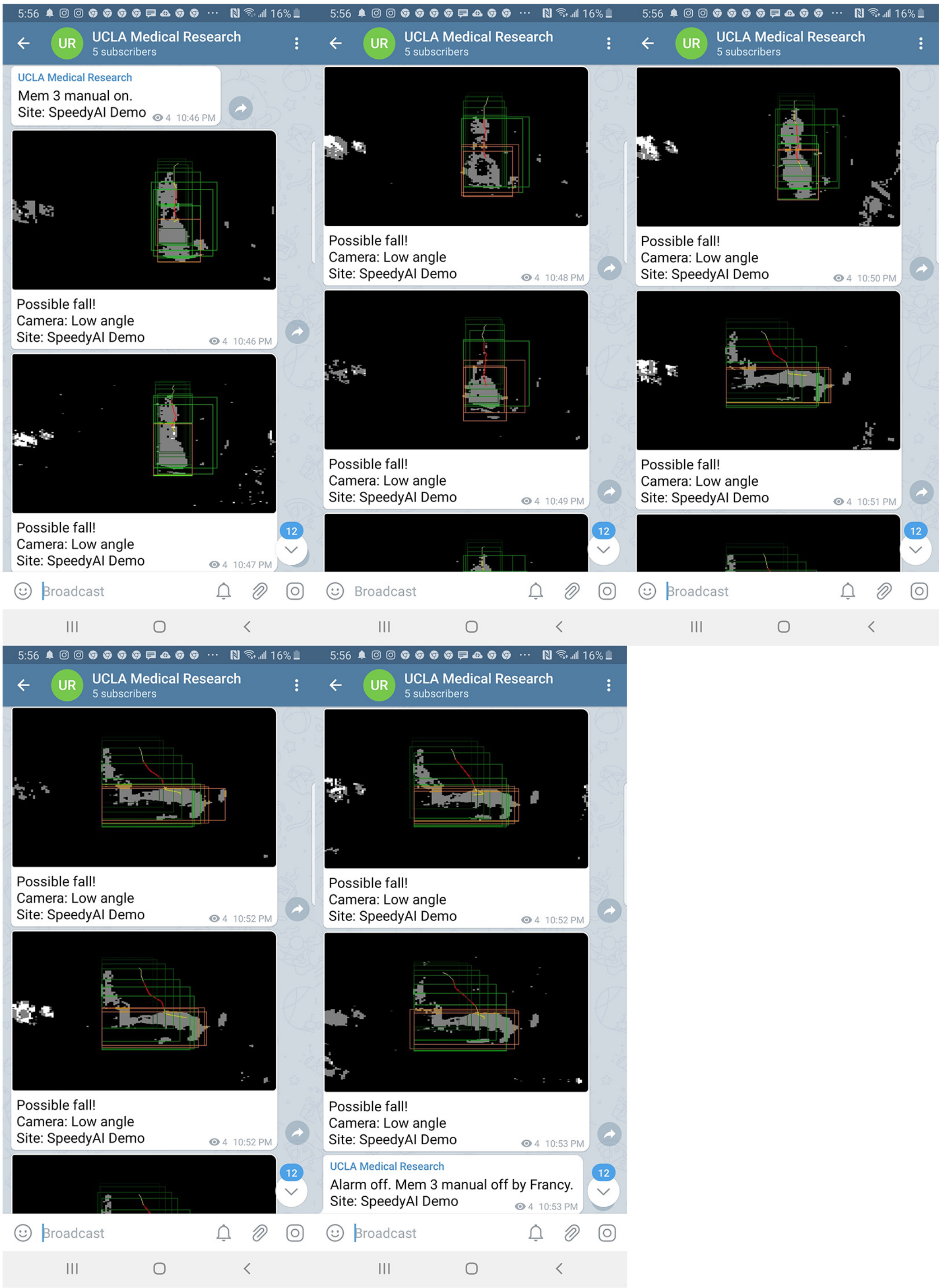

s, Stumbling test along the screen edge

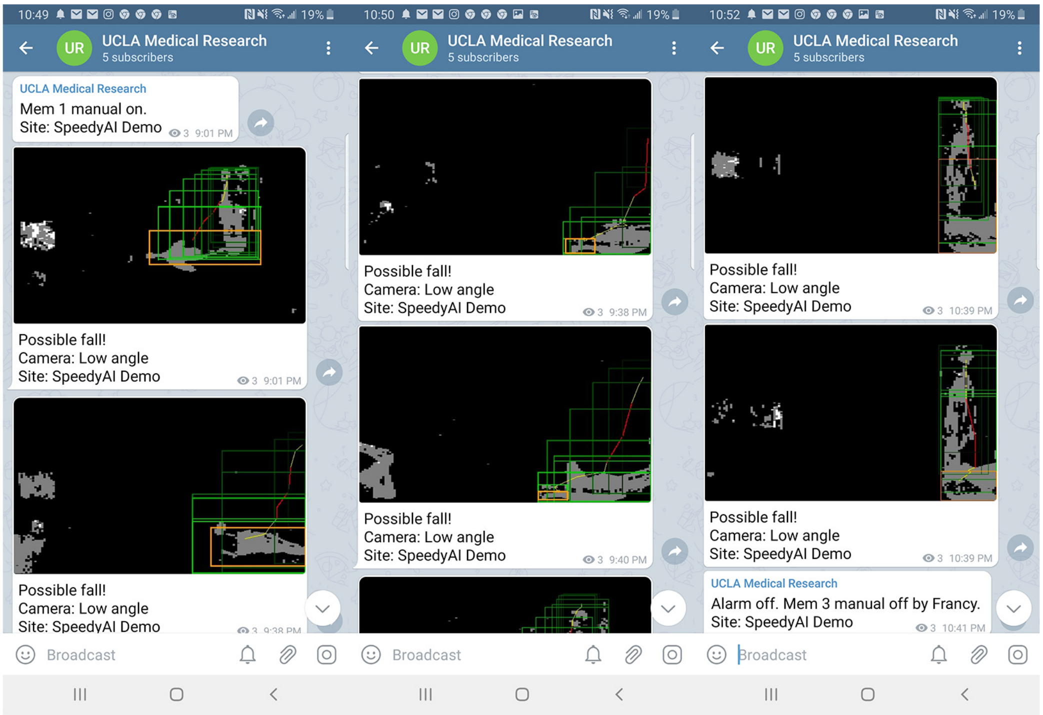

t, Stumbling test in the presence of multiple moving people/objects on Camera 1 and Camera 2 (Part 1)

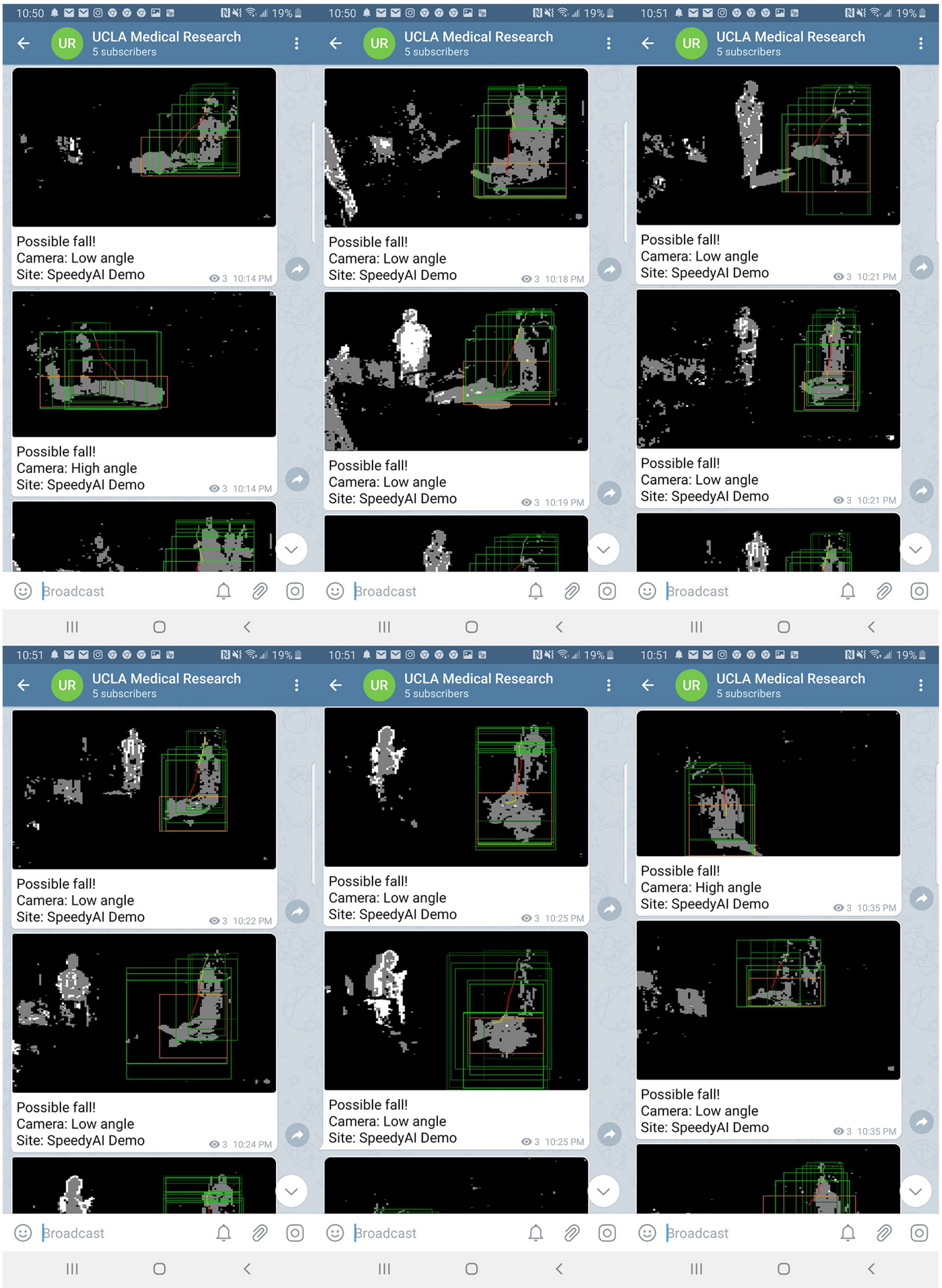

u, Stumbling test in the presence of multiple moving people/objects on Camera 1 and Camera 2 (Part 2)

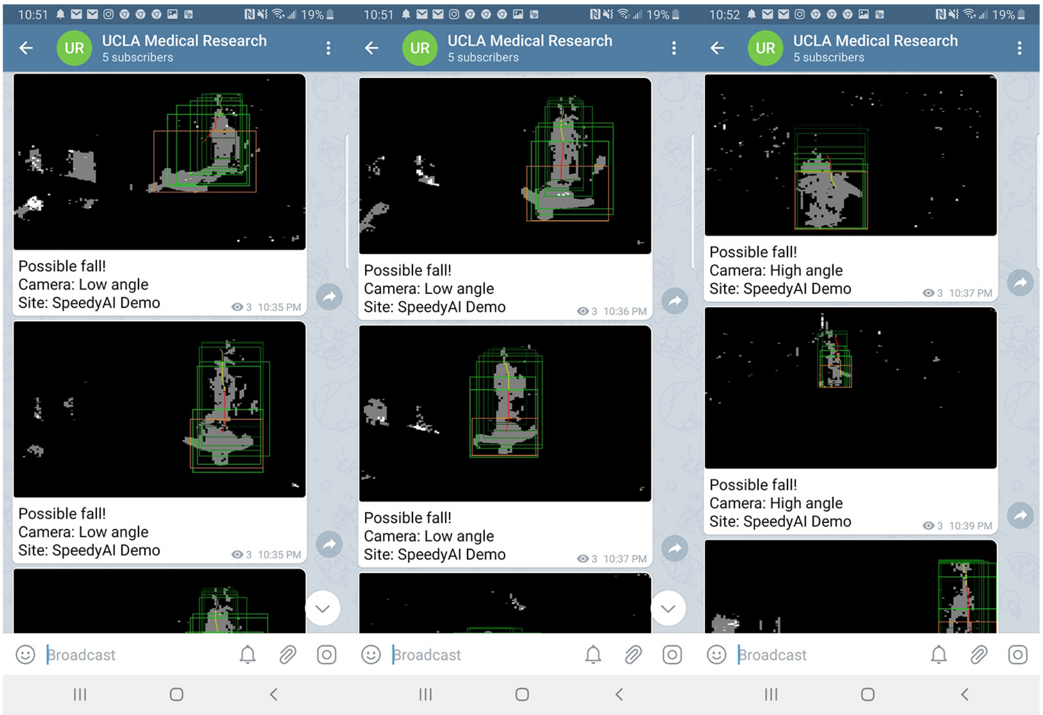

v, Fall test behind a transparent glass window and opened mini-blinds

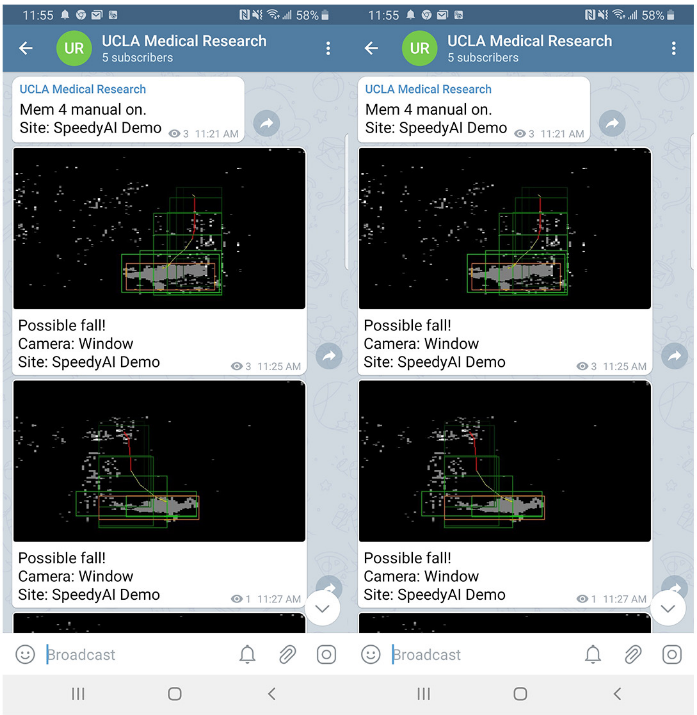

w, Other random fall tests

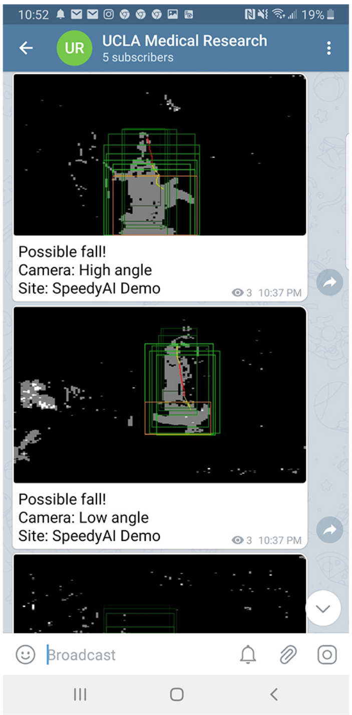

A 2.5 meter-high camera (Cam 2) capturing a fall at a very steep downward angle

A shoulder-height camera (Cam 1) capturing the same fall

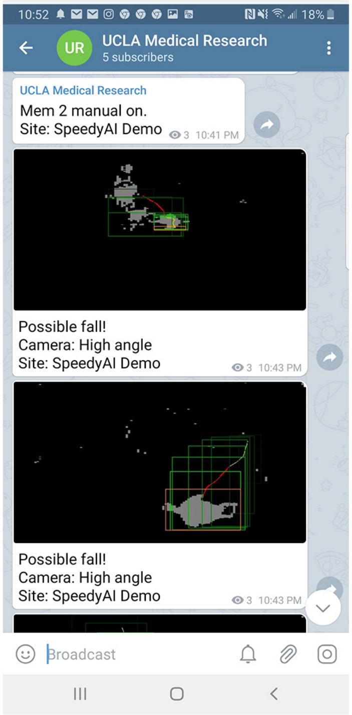

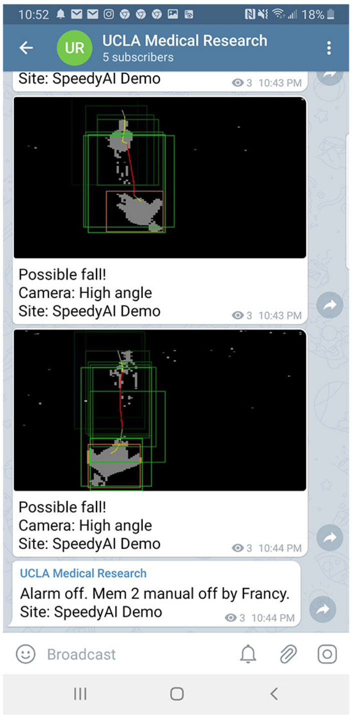

Falling off a ladder captured by Cam 2

Jumping down from a desk and falling captured by Cam 2
